# Syngeneic mouse model of human HER2+ metastatic breast cancer for the evaluation of trastuzumab emtansine combined with oncolytic rhabdovirus

**DOI:** 10.3389/fimmu.2023.1181014

**Published:** 2023-04-19

**Authors:** Zaid Taha, Mathieu J.F. Crupi, Nouf Alluqmani, Faiha Fareez, Kristy Ng, Judy Sobh, Emily Lee, Andrew Chen, Max Thomson, Marcus M. Spinelli, Carolina S. Ilkow, John C. Bell, Rozanne Arulanandam, Jean-Simon Diallo

**Affiliations:** ^1^ Centre for Cancer Therapeutics, Ottawa Hospital Research Institute, Ottawa, ON, Canada; ^2^ Department of Biochemistry, Microbiology, and Immunology, Faculty of Medicine, University of Ottawa, Ottawa, ON, Canada; ^3^ Department of Pathology and Molecular Medicine, McMaster University, Hamilton, ON, Canada

**Keywords:** mouse model, HER2, immunotherapy, oncolytic virus, VSVΔ51, antibody-drug conjugate, Kadcyla^®^, trastuzumab

## Abstract

**Background:**

Established mouse models of HER2+ cancer are based on the over-expression of rodent Neu/Erbb2 homologues, which are incompatible with human HER2 (huHER2) targeted therapeutics. Additionally, the use of immune-deficient xenograft or transgenic models precludes assessment of native anti-tumour immune responses. These hurdles have been a challenge for our understanding of the immune mechanisms behind huHER2-targeting immunotherapies.

**Methods:**

To assess the immune impacts of our huHER2-targeted combination strategy, we generated a syngeneic mouse model of huHER2+ breast cancer, using a truncated form of huHER2, HER2T. Following validation of this model, we next treated tumour-bearing with our immunotherapy strategy: oncolytic vesicular stomatitis virus (VSVΔ51) with clinically approved antibody-drug conjugate targeting huHER2, trastuzumab emtansine (T-DM1). We assessed efficacy through tumour control, survival, and immune analyses.

**Results:**

The generated truncated HER2T construct was non-immunogenic in wildtype BALB/c mice upon expression in murine mammary carcinoma 4T1.2 cells. Treatment of 4T1.2-HER2T tumours with VSVΔ51+T-DM1 yielded robust curative efficacy compared to controls, and broad immunologic memory. Interrogation of anti-tumour immunity revealed tumour infiltration by CD4+ T cells, and activation of B, NK, and dendritic cell responses, as well as tumour-reactive serum IgG.

**Conclusions:**

The 4T1.2-HER2T model was used to evaluate the anti-tumour immune responses following our complex pharmacoviral treatment strategy. These data demonstrate utility of the syngeneic HER2T model for assessment of huHER2-targeted therapies in an immune-competent *in vivo* setting. We further demonstrated that HER2T can be implemented in multiple other syngeneic tumour models, including but not limited to colorectal and ovarian models. These data also suggest that the HER2T platform may be used to assess a range of surface-HER2T targeting approaches, such as CAR-T, T-cell engagers, antibodies, or even retargeted oncolytic viruses.

## 1 Introduction

Immunotherapeutic studies of human HER2+ cancer are limited by available mouse models, which include transgenic *MMTV*-*erbb2* and *MMTV*-*neu* (1, 2). However, both of these models express rodent homologues of HER2 and are therefore not ideal for the evaluation of anti-human HER2 biologics (3). The documented spontaneity and latency (4, 5) of tumourigenesis in these MMTV-based HER2 transgenic models also make them impractical for rapidly testing and optimizing more complex regimens (e.g. combination therapies), or for evaluating HER2-targeted therapies in other HER2-overexpressing malignancies with divergent underlying oncogenic drivers (e.g. gastric cancer, ovarian cancer). While syngeneic models would be more convenient and versatile, full-length human HER2 (huHER2) is highly immunogenic in mice (6–8) thereby causing strong anti-HER2 immune responses that confound the evaluation of anti-neoplastic immunotherapies.

Trastuzumab (Herceptin^®^) is a mainstay in the treatment of HER2+ breast cancer, and is also approved for gastric cancer. As the first clinically-approved HER2-targeted antibody-drug conjugate (ADC), trastuzumab emtansine (Kadcyla^®^, T-DM1) demonstrated clinical superiority in the treatment of HER2+ metastatic breast cancer (mBC), including trastuzumab-resistance mBC. However, the activity of Kadcyla is limited and could be further improved. While altering the ADC payload is one approach to increase activity (9), it is fundamentally limited to HER2-expression in tumours, which may be heterogenous. An alternative approach is combination with complementary modalities that are not HER2-dependent.

While not yet approved for mBC, oncolytic viruses (OV) have made some clinical progress (10), with the first OV approved for the treatment of melanoma (11) and more recently glioblastoma (12). While the mechanism of OVs partially relies on direct oncolysis of the tumour, OVs are potent stimulants of anti-tumour immunity through engagement and redirection of innate and adaptive responses. However, heterogeneity of tumour infection by OVs and subsequent treatment response remains a major challenge.

Our previous work demonstrated the utility of T-DM1 as a targeted enhancer of oncolytic vesicular stomatitis virus (VSVΔ51) in HER2-amplified cancers (13), as a result of mechanistic synergy between microtubule destabilizing agents and VSVΔ51 (14). In that work, we demonstrated improvement in survival in a xenograft model of trastuzumab-resistant HER2+ breast cancer following combination treatment with VSVΔ51+T-DM1. However, xenograft models do not adequately recapitulate pro-inflammatory properties of VSVΔ51, nor allow assessment of its interaction with potentially immune-modulatory activities of T-DM1.

To overcome the limitations of xenograft models in assessing immunotherapies such as VSVΔ51+T-DM1, we set out to develop a syngeneic immune-competent model employing truncated human HER2 (HER2T). We hypothesized that a minimal HER2 construct retaining the trastuzumab epitope without kinase signaling activity may allow for the generation of an accessible and practical syngeneic model that elicits less immunogenicity against huHER2. This model would permit the use of murine cancer cell lines of various origins, including mBC. We further considered that this model could provide utility for investigation of a range of more complex HER2-targeted therapeutics.

Herein, we developed and characterized an immune-competent mouse model of human HER2+ mBC using HER2T expressed in murine 4T1.2 mBC cells. We then used this model to profile the therapeutic effects and anti-tumour immune responses of VSVΔ51+T-DM1 combination revealing remarkable efficacy, even following systemic delivery, associated with both HER2-specific and non-specific immune-responses.

## 2 Materials and methods

### 2.1 Cells

Vero (CCL-81), CT26WT (CRL-2638), HEK293T cells (CRL-3216) were acquired from ATCC and cultured in Dulbecco’s modified Eagle’s medium (DMEM, HyClone, Waltham, Massachusetts or Corning, Manassas, Virginia) with 10% FBS. JIMT-1 (ACC589) were acquired from DSMZ and cultured in DMEM with 10% FBS. 4T1.2 cells were cultured in Roswell Park Memorial Institute medium (RPMI; ATCC, Cat. # ATCC 30-2001) with 10% FBS. NK-92MI (CRL-2408) cells were cultured in RPMI with 10% FBS, 30 mM HEPES, 50 mM 2-mercaptoethanol (ThermoFisher Scientific, Cat. # 21985023).

ID8 cells were cultured in DMEM supplemented with 4% FBS and 1% ITSS (5 μg/mL insulin, 5 μg/mL transferrin, and 5 ng/mL sodium selenite; R&D Systems, MN, USA, Cat. # AR013). MC38 cells were cultured in DMEM with 10% FBS. AF2068 cells were cultured in RPMI with 10% FBS.

All media were supplemented with 1% penicillin-streptomycin solution (ThermoFisher Scientific, Cat. # 15140163). All cells were maintained at 37^0^C in a 5% CO_2_ humidified incubator, routinely tested for mycoplasma contamination by Hoechst staining and PCR (Diamed, Mississauga, Ontario, Catalogue # ABMG238) and used within 3-10 passages since thaw.

### 2.2 Plasmids

#### 2.2.1 pLenti-PGK-CD16a-Blast

The coding sequence of human wildtype CD16a (NM_001127593.1) was cloned into the pLenti PGK Blast V5-LUC (w528-1) plasmid, which was a gift from Eric Campeau & Paul Kaufman (Addgene plasmid # 19166; http://n2t.net/addgene:19166; RRID : Addgene_19166). 5’ SalI and 3’ XbaI restriction cut sites were utilized to replace the *V5-LUC* construct with *CD16a*.

#### 2.2.2 pLenti-CMV-HER2T-Puro

The expression plasmid encoding human wildtype *ERBB2* (NM_004448.3) was obtained as a gift from Mien-Chie Hung (Addgene plasmid # 16257; http://n2t.net/addgene:1625;RRID : Addgene16257). The required truncations were generated and cloned into the pLenti-CMV-EGFP-Puro plasmid, which was a gift from Eric Campeau & Paul Kaufman (Addgene plasmid # 17448; http://n2t.net/addgene:17448;RRID : Addgene_17448). 5’ XbaI and 3’ SalI restriction cut sites were utilized to replace the *EGFP* construct with *HER2T*.

#### 2.2.3 Lentivirus packaging plasmids

psPAX2 (gag-pol; psPAX2 was a gift from Didier Trono (Addgene plasmid # 12260; http://n2t.net/addgene:12260; RRID : Addgene_12260) and pMD2.G (VSV-G envelope; pMD2.G was a gift from Didier Trono (Addgene plasmid # 12259; http://n2t.net/addgene:12259; RRID : Addgene_12259) were used for subsequent lentiviral rescue.

### 2.3 Oncolytic vesicular stomatitis virus

The oncolytic versions of VSV (Indiana serotype) encoding a firefly luciferase protein tag (VSVΔ51-Fluc), green fluorescent protein (VSVΔ51-GFP) tag, TNFα, shPD-L1, or interleukin-12 were grown and titered on Vero cells as previously described (15, 16). Briefly, VSVΔ51 was added at an MOI of 0.01 to 95% confluent Vero cells in 150 mm culture dishes or roller bottles in a total volume of 25 ml complete DMEM. Inoculated Vero cells were incubated at 37°C with 5% CO_2_ for 24 hours or until approximately 50% CPE (cytopathic effects, cell rounding) was observed. Supernatants were collected and pelleted at 780 *x g* to clear heavy debris. Virus contained within the cleared supernatant was subsequently subject to 0.22 µm membrane filtration and purified using 5-50% Optiprep (Sigma-Aldrich, Oakville, ON, Canada, Cat. # D1556) gradient. The purified virus suspension was aliquoted and frozen at -80^0^C. For all virus infections, viruses were diluted in serum free DMEM to obtain the specified multiplicity of infection (MOI), or for mock infection cells were supplemented with an equal volume of serum free DMEM. For high throughput luciferase titering (15), Vero cells were prepared to be 95-100% confluent in opaque white 96-well plates in 100 μl complete DMEM supplemented with 30 mM HEPES. VSVΔ51-Fluc infected samples to be titered were transferred (25 µl/well) onto the Vero cells along with a standard curve prepared from a purified virus stock of known titer and diluted from 10^8^ PFU/ml – 10^1^ PFU/ml in duplicate for each 96-well plate. Vero plates were then incubated for 5 hours at 37°C 5% CO_2_, following which a D-luciferin (PerkinElmer, Waltham, MA, USA, Cat. # 122799) solution was prepared (2 mg/ml in sterile PBS). Following priming of the Biotek Synergy microplate reader, plates were inserted into the instrument and the D-luciferin solution was automatically dispensed at 25 µl/well. Luminescence was read at an appropriate fixed sensitivity. Standard curve values allow for the generation of a Hill equation which was applied to the titered samples to obtain Viral Expression Units (VEU) using R software. For virus titration using standard plaque assay, Vero cells were seeded into 12-well plates at a final density of 3E5 cells per well. Infectious supernatants were serially diluted using serum-free DMEM, transferred (500 µL/well) onto Vero cells and incubated at 37°C, 5% CO_2_ for 45 minutes, following which media was removed and replaced with 1ml/well of an agarose overlay (1:1 ratio of 1% agarose mixed with 2X DMEM containing 20% FBS). After a 24 hour incubation, plaques were fixed with methanol: glacial acetic acid in a 3:1 ratio for a minimum of 1 hour, then stained for 30 minutes with a Coomassie Blue solution (Sigma, Cat. # B0149; 4 g Coomassie Brilliant Blue R, 800 ml methanol, 400 ml acetic acid and 2800 ml distilled water) to visualize and count plaques.

### 2.4 Viral plaque expansion assay

For quantification of viral spread, cells were seeded in 6-well plates for overnight confluence, then treated and infected as described then overlaid with a 0.5% agarose-DMEM plug. Wells were imaged using the Cellomics Arrayscan (if VSVΔ51-GFP was used) at 24-72hpi, then fixed (3:1 acetic acid:methanol) and stained with Coomassie blue. Plaque diameters were quantified using ImageJ software.

### 2.5 Viral growth curves

Cells were seeded in 24 well plates for overnight confluency. Cells were then inoculated with VSVΔ51-Fluc at an MOI of 0.01 (multi-step growth curve) or 1.0 (single-step growth curve).

Cells infected at MOI 1.0 were incubated for 60 minutes, following by washing and replenishing with fresh medium. Cells were incubated up to 48 hpi, with 200 µl of supernatant collected and frozen at -80^0^C at the following timepoints: 0, 4, 8, 12, 24, 32, 48 hpi. Viral titer in collected supernatant was quantified by high-throughput titering as previously described.

### 2.6 Lentivirus preparation and transduction

Lentiviruses were generated using 3^rd^ generation transfer plasmids. Lentiviral rescue was performed by co-transfection of 293T cells, in 150 mm dishes, with psPAX2:PMD2.G:pLenti transfer at a molar ratio of 2:1:3 using PolyJet DNA transfection reagent (SignaGen Laboratories, MD, USA, Cat. # SL100688) according to manufacturer’s protocol. Lentiviral supernatant was filtered through 0.45 µm membrane, and added to target cells with 8 µg/ml polybrene (Millipore-Sigma, Cat. # 1003) and overnight incubation at 37^0^C. Target cells underwent antibiotic selection for 7-14 days before validation and expansion.

### 2.7 Drugs, antibodies, cytokines

Trastuzumab (Herceptin^®^; Hoffman-La Roche, Mississauga, Ontario, Canada), T-DM1 (Kadcyla^®^; trastuzumab emtansine; Hoffman- La Roche) and IVIG (Gamunex^®^; 10% immune globulin intravenous (human), Grifols, Mississauga, Ontario, Canada, DIN 02247724) were obtained from clinical preparations at the Ottawa Hospital Pharmacy, stored at 4°C and used at the indicated concentrations. Human (R&D Systems, Cat. # 236-EG) or mouse (R&D Systems, Cat. # 2028-EG) recombinant EGF were used to stimulate serum-starved cells for 30 minutes prior to protein extraction.

### 2.8 Cell-based assays

Metabolic activity of cells was assessed using alamarBlue (Invitrogen, MA, USA, Cat. # DAL1025) or resazurin sodium salt (Sigma-Aldrich, Cat. # R7017) according to the manufacturer’s protocol. Fluorescence was measured at 590 nm upon excitation at 530 nm using the BioTek Synergy or Cytation 5 Microplate Readers (BioTek, VT, USA). To assess cell proliferation, cells were seeded at 1 x 10^4^ cells per well in 24-well plates. Three wells were trypsinized and counted daily, for 7-10 days, using the Countess automated cell counter (Invitrogen, Cat. # AMQAF1000). Medium was changed on all remaining wells daily. For DNA transfections, cells were transfected at 70-80% confluency using PolyJet DNA transfection reagent (SignaGen) according to the manufacturer’s protocol. Cells were treated then harvested 48 hours-post transfection.

### 2.9 Antibody internalization assay

Target cells were seeded in 96-well plates at 5-10 x 10^4^ cells/well in 50 ul complete medium. Trastuzumab was used as the primary antibody for internalization, using the Incucyte^®^ Fabfluor-pH Red Antibody Labeling Dye (Sartorius, Goettingen, Germany, Cat. # 4722) according to the manufacturer’s protocol.

### 2.10 Cell lysis and western blotting

Whole cell lysates were obtained by lysing cells in 50 mM HEPES, pH 7.4, 150 nM NaCl, 2 mM Na_3_VO_4_, 10 mM EDTA, 100 mM NaF, 10 mM Na_2_P_2_O_7_, protease/phosphatase inhibitor cocktail (Cell Signaling Technology (CST), MA, USA, Cat. # 5872) and 1% Triton X-100 on ice. Protein concentration was determined by bicinchoninic acid assay (Thermo Scientific, Cat. # 23225) and 10-20 µg of cell extract were resolved using the NuPAGE SDS-PAGE system (Invitrogen, Carlsbad, CA, USA, Cat. # NP0322) and transferred onto a nitrocellulose membrane (BioRad, Cat. # 1620115). Membranes were blocked with 5% milk in 1x TBS-T and probed with a rabbit polyclonal antibody to β-actin (1:5000, CST, Cat. # 49705) as a loading control, or phospho-EGFR (1:1000, CST, Cat. # #3777), total EGFR (1:1000, CST, Cat. # 4267), phospho-ERK1/2 (1:2000, CST, Cat. # 4370), total ERK1/2 (1:1000, CST, Cat. # 4695), or HER-2/Erbb2 (1:1000, Invitrogen, Cat. # MA5-13105) followed by incubation with horseradish-peroxidase conjugated rabbit or mouse secondary antibodies (1:5000), respectively (Jackson ImmunoResearch Laboratory, West Grove, PA, USA, Cat. # 711-035-152 (anti-rabbit) 715-035-150 (anti-mouse)). Supersignal West Pico Plus Chemiluminescent substrate (Thermo Scientific, Burlington, Ontario, Canada, Cat. # 34577) was used to visualize the protein bands.

### 2.11 Single cell sorting

Single cell suspensions of HER2T-expressing cells were prepared for sorting, at 1-2 x 10^6^ cells/ml. Monoclones (1 cell per well) and polyclones (50 cells per well) were sorted into complete medium using the Sony MA900 (Sony Biotechnology, CA, USA). Cells were grown for 5-7 days in selection medium, followed by HER2T quantification by flow cytometry. All populations were validated as described, and representative populations were selected for subsequent *in vitro* and *in vivo* testing. Only polyclonal populations were used in the data of this manuscript to avoid the clonal variability observed in monoclonal populations.

### 2.12 Antibody-dependent cell-mediated cytotoxicity (ADCC) assay

Target cells were seeded for overnight confluence in 24-well plates. The next day, target cells were treated with trastuzumab (10 µg/ml), mouse serum (1:100), or mock in serum free medium, at 4^0^C for 30 minutes. Co-cultures with NK-92MI CD16a+ cells were performed at a ratio of 5:1 (E:T ratio), and incubated for 12-16 hours. All wells were aspirated and washed to remove effector and dead cells. Remaining target cells were stained with DAPI (1:5000) and imaged. For quantification of cell death, leftover target cells were then fixed and stained with 3:1 acetic acid:methanol fixative with Coomassie Blue for 60 minutes; dye was solubilized using 1% SDS in PBS and absorbance was read at 595 nm.

### 2.13 Immunofluorescence staining

Cells on sterile glass coverslips were incubated overnight at 37°C. Cells were then fixed with 4% PFA, quenched with 100 mM glycine in PBS* (supplemented with 1 mM CaCl_2_ and 0.5 mM MgCl_2_), and blocked in 5% BSA-PBS*. For detection of cell surface HER2 or HER2T, slides were incubated overnight at 4°C with trastuzumab (1:2000), in 1% BSA-PBS*. Coverslips were washed then incubated for 1 h at room temperature with a goat anti-human IgG conjugated to Alexa594 (Invitrogen, Cat. # A-11014, 1:200). Coverslips were mounted on slides with ProLong Gold Antifade reagent with DAPI (Life technologies/Thermo Scientific, Burlington, Ontario, Cat. # P36930) and stored at 4°C. Images were taken using the AxioCam HRm camera (Carl Zeiss Ltd, Toronto ON) mounted on the Zeiss Axioscope Imager M1 microscope.

### 2.14 Immunohistochemistry

Following harvest, tumour samples fixed in 10% formalin (Sigma, Cat. # HT501128) then transferred to a 70% EtOH solution for storage until sectioning and paraffin embedding. Mounted sections were deparaffinized, hydrated, and stained with H&E. After washing, slides were dehydrated through alcohol and xylene, followed by mounting with Permount (VWR, Ontario, Canada, Cat. # 100496-550) and coverslips. After drying, slides were cleaned, scanned using the ZEISS Axio Scan.Z1 (20x magnification; Carl Zeiss Canada Ltd.), and delivered to a pathologist for blinded scoring and histological assessment.

### 2.15 Immune profiling

#### 2.15.1 Tissue processing

tumours were dissociated using the Miltenyi mouse tumour dissociation kit (Miltenyi Biotec, CA, USA, Cat. # 130-096-730) and the gentleMACS Octo Dissociator (Miltenyi Biotec, Cat. # 130-096-427). Spleens and TdLN (ipsilateral axillary, inguinal, cervical) were collected and dissociated by crushing the organs through a 70 µm strainer using the plunger of a 3 mL syringe. All dissociated spleens underwent erythrocyte lysis using ACK buffer (Gibco, Cat. # A1049201). All cell suspensions were counted; 2 x 10^6^ cells were resuspended in 200 µl of FACS buffer (0.5% BSA-PBS) and transferred to round-bottom 96-well plates for staining. Serum was collected from mice using Microvette tubes (Sarstedt, Nümbrecht, Germany, Cat. # 20.1291), and clarified by centrifugation at 10,000 x g for 5 minutes at room temperature.

#### 2.15.2 Flow cytometry

After processing tissues as described above, cells were stained with fixable viability dye FVS510 (1:1000, BD Biosciences, NJ, USA, Cat. #564406) in PBS for 30 minutes at room temperature. Cells were washed and then incubated with anti-CD16/32 (1:100, BD Biosciences, Cat. # 553141) in 0.5% BSA-PBS for 30 minutes at 4^0^C to block non-specific antibody interaction with F_c_ receptors. Cells were subsequently stained with a subset of antibodies against the following targets: CD45-BV786 (1:1000, BD Biosciences, Cat. # 564225), CD3-AlexaFluor 700 (1:100, BD Biosciences, Cat. # 561805), CD4-V450 (1:1000, BD Biosciences, Cat. # 560468), CD8a-PerCP-Cy5.5 (1:100, BD Biosciences, Cat. #551162), CD8a-PE-CF594 (1:100, BD Biosciences, Cat. # 562283), CD25-PE (1:100, Thermo Scientific, Cat. # 12-0251-82), CD69-BV605 (1:100, BD Biosciences, Cat. #563290), CD44-APC (1:100, BD Biosciences, Cat. #563058), CD62L-FITC (1:100, BD Biosciences, Cat. #553150), PD-1-APC (1:100, BD Biosciences, Cat. # 562671), CD127-PE-Cy7 (1:100, BD Biosciences, Cat. # 560733), CD49b-FITC (1:100, BD Biosciences, Cat. # 553857), CD122-PE (1:100, Invitrogen, Cat. # 12-1221-82), CD11b-APC (1:200, BD Biosciences, Cat. #553312), CD11c-PE (1:100, BD Biosciences, 553802), CD86-APC-R700 (1:100, BD Biosciences, Cat. # 565479), IA/IE-BV605 (1:200, BD Biosciences, Cat. # 563413), CD19 (1:100, BD Biosciences, Cat. # 553785), F4/80-APC-Cy7 (1:100, BioLegend, CA, USA, Cat. #123118), trastuzumab (1:1000) and PE-conjugated goat anti-human secondary IgG (1:500, Invitrogen, Cat. # PA1-86078), MHC class I H 2Kd-APC (1:300, Thermo Scientific, Cat. # 17-5957-80), PD-L1-APC-Cy7 (1:100, BioLegend, Cat. # 124313). Cells were then washed and resuspended in 1% paraformaldehyde (PFA) in PBS. Samples were acquired with the BD LSRFortessa™, BD FACSCelesta™, and the Cytel^®^ Aurora spectral cytometer at the University of Ottawa Flow Cytometry and Virometry core facility (Director: Dr. Vera Tang), and the Ottawa Hospital Research Institute Flow Cytometry and Cell Sorting core facility (Director: Fernando Ortiz). Data were analyzed using FlowJo v10.8 software. Dimensionality reduction t-SNE plots were generated using the opt-SNE (17) learning configuration and the FIt-SNE (18) algorithm. Unstained controls, and fluorescence-minus-one (FMO) controls were prepared in parallel. Ultracomp eBeads (Thermo Scientific, Cat. # 01-2222-42) single-stained beads were used for compensation.

#### 2.15.3 IgG assay

3 x 10^5^ cells were resuspended in 100 µl of 0.5% BSA-PBS and transferred to a round-bottom 96-well plate. Cells were incubated with mouse serum (1:100) for 60 minutes at 4^0^C, then incubated with goat anti-mouse IgG-AlexaFluorPlus-488 (1:300, Invitrogen, Cat. # A32723). Cells were washed and resuspended in 1% PFA-PBS, then acquired by flow cytometry.

#### 2.15.4 Cytokine and chemokine assay

For detection of serum cytokines and chemokines, serum was pooled from n = 5 mice (4T1.2 or 4T1.2-HER2T tumour bearing mice) and used undiluted for processing and subsequent probing of the cytokine array dot blot (R&D Systems, Cat. # ARY006) according to the manufacturer’s instructions. Quantitative densitometry was performed using ImageJ software.

#### 2.15.5 Mouse IFN-γ single-color enzyme-linked immune absorbent spot (ELISPOT)

Immunospot ELISPOT plates were prepared according to manufacturer’s protocol (Immunospot, OH, USA, Cat. # mIFNgp-1M/5), with stimulants added to wells as indicated. 2 x 10^5^ splenocytes or peripheral blood lymphocytes incubated in CTL medium in the IFN-γ pre-coated plates. Spots were counted using ImmunoSpot^®^ Software version 5.3. Counts from unstimulated/DMSO control were treated as background and subsequently subtracted from all conditions within the same sample. Stimulants used were: DMSO, 4T1.2 lysates, 4T1.2-HER2 lysates, irradiated CT26-HER2T cells, irradiated 4T1.2 cells, 10 µM VSV-N peptide, or 5 µM PMA/Ionomycin. Lysates were produced by 3x freeze-thaw of 2 x 10^6^ cells per 100 µl of CTL medium. CT26-HER2T and 4T1.2 cells were resuspended in CTL medium at 2 x 10^6^ cells per 100 µl, then irradiated (Pantak HF320 X-Ray Machine) with 60 Gy or 90 Gy, respectively.

### 2.16 Syngeneic models

Six to eight week old female BALB/c (Charles River Laboratories, MA, USA, Strain code #028) or C57BL/6 (Strain code #027) were used throughout the study. Cells were implanted as indicated in figures, with or without cold Geltrex^®^ (1:1 ratio, Thermo Fisher, Cat. # A1413201). For palpable tumours, mice were treated when tumours reached 80-100 mm^3^ as indicated in figures. Mice were culled when tumour volumes reached above 1500 mm^3^, and according to the institutional guidelines review board for animal care. For 4T1.2 lung metastasis model, lungs were harvested, stained, and counted as previously described (19). All rechallenge studies were performed D90 post-implantation, by implanting cells at double the original seeding density and in the contralateral side. Intraperitoneal ID8*Tp53-/–*HER2T-Fluc tumour progression was monitored by IVIS imaging of animals. Mice were injected with D-luciferin at 10 mg/ml (PerkinElmer, MA, USA, Cat. # 122799-5), and luminescence was read at an exposure time of 30s and 60s. Total flux was analyzed using LivingImage v4.7 software. Palpable tumours (s.c. or o.t.) were monitored by regular measurement using electronic calipers, and volumes were calculated as (length x width^2^)/2.

### 2.17 Quantitative real-time PCR

Frozen tumours were crushed using a dounce homogenizer. Cells and tumours were collected and RNA extraction was performed by using QiaShredder columns (Qiagen, Hilden, Germany, Cat. # 79656) and RNeasy kits (Qiagen, Cat. # 74106). RNA was converted to cDNA using (Thermo Fisher Scientific, Cat. # K1622). Real-time PCRs were performed according to the manufacturer’s protocol (Thermo Fisher Scientific, Cat. # A25776) on a 7500 Fast Real-Time PCR system (Applied Biosystems). Optimal thresholds, reaction efficiencies, and *C*_t_ values were determined using the ABI software and melt curves for each primer exhibited a single peak. Gene expression relative to *HPRT* was calculated by the Pfaffl method. Fold-change was determined relative to the Mock control for each gene.

**Primers**

HER2T F: ATCATCTCTGCGGTGGTTG

HER2T R: CGTGTACTTCCGGATCTTCTG

mHPRT F: TGAAGAGCTACTGTAATGATCAGTCAA

mHPRT R: AGCAAGCTTGCAACCTTAACCA

### 2.18 Statistics and reproducibility

All graphs and statistical analyses were performed using Excel or GraphPad Prism v.9. Individual statistical tests were detailed in figure legends. One-way or two-way ANOVA were followed-up with Fisher’s LSD test when few planned comparisons were pre-determined. Normal distribution of the data was assessed using D’Agostino & Pearson omnibus and Shapiro-Wilk normality tests. Non-parametric tests were implemented if assumptions for parametric tests were not met. Alpha levels for all tests were 0.05 (confidence levels of 95%). Analysis of *in vivo* survival data was performed by the Kaplan-Meier method followed by log-rank test. *In vivo* group sizes were based on a power calculation of 0.8, with an anticipating halving of mOS. Biological replicates are indicated by a number n, and defined as per NIH guidelines, and error calculated as the standard error of the mean (SEM).

### 2.19 Declarations

#### 2.19.1 Ethics

*In vivo* experiments were performed *via* protocols OHRI-2265 and OHRI-2264 which are in good standing with the Animal Care Committee, and care and treatment of animals was in accordance with the ethical standards of the Canadian Council on Animal Care and with the Animals for Research Act.

#### 2.19.2 Data availability

All relevant data are available from the authors upon request to the corresponding author.

## 3 Results

### 3.1 4T1.2 cells expressing HER2T bind to trastuzumab *in vitro*

**Figure 1:**
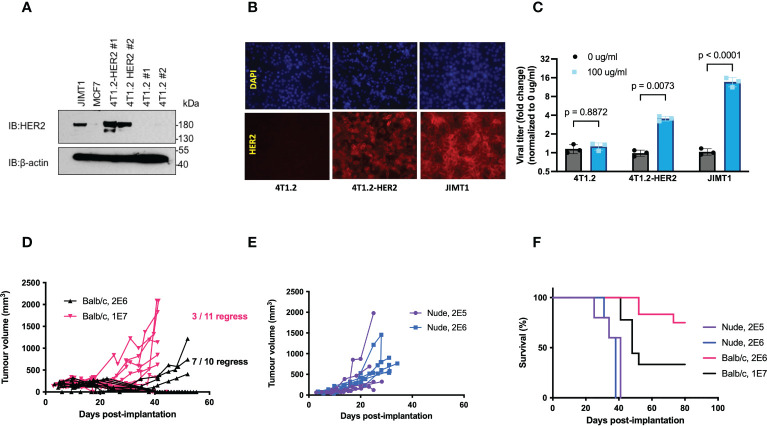
Expression of full length hHER2 promotes tumour rejection in immunocompetent BALB/c mice. **(A)** Western blots of whole cell lysates from JIMT1, MCF7, and two clones of 4T1.2-HER2 and 4T1.2 probed with anti-HER2 antibody and β-actin as loading control. **(B)** 4T1.2, 4T1.2-HER2, and JIMT1 cells were seeded on glass coverslips, stained with trastuzumab (1:1000), and visualized using a goat anti-human IgG 2^0^ antibody conjugated to Alexa Fluor-594 (20x magnification). **(C)** 4T1.2, 4T1.2-HER2, and JIMT1 cells were treated with PBS or T-DM1 at the indicated concentration for 4 hours, then infected with VSVΔ51-Fluc at MOI 0.001; viral output was quantified 48 hpi (mean ± SEM; n = 3; two-way ANOVA with Fisher’s LSD test) **(D, E)** 4T1.2-HER2 cells were implanted in **(D)** athymic nude mice or **(E)** wildtype BALB/c mice at the indicated cell numbers, subcutaneously in the right flank. Tumour volume was monitored by measurements using electronic calipers, and **(F)** overall survival was tracked.

We first confirmed that the expression of full length HER2 in 4T1.2 cells (4T1.2-HER2 (20)) enabled increased VSVΔ51 spread following 4 hr pretreatment with T-DM1 to a similar extent as in JIMT1 human HER2+ breast cancer cells (**Figures 1A–C**). However, implantation of 4T1.2-HER2 cells in wildtype BALB/c mice led to frequent tumour rejection (27% rejection at 1 x 10^7^ cells, 70% rejection at 1 x 10^6^ cells), but not in athymic nude mice (**Figures 1D–F**), confirming substantial immunogenicity of the full length huHER2.

**Figure 2:**
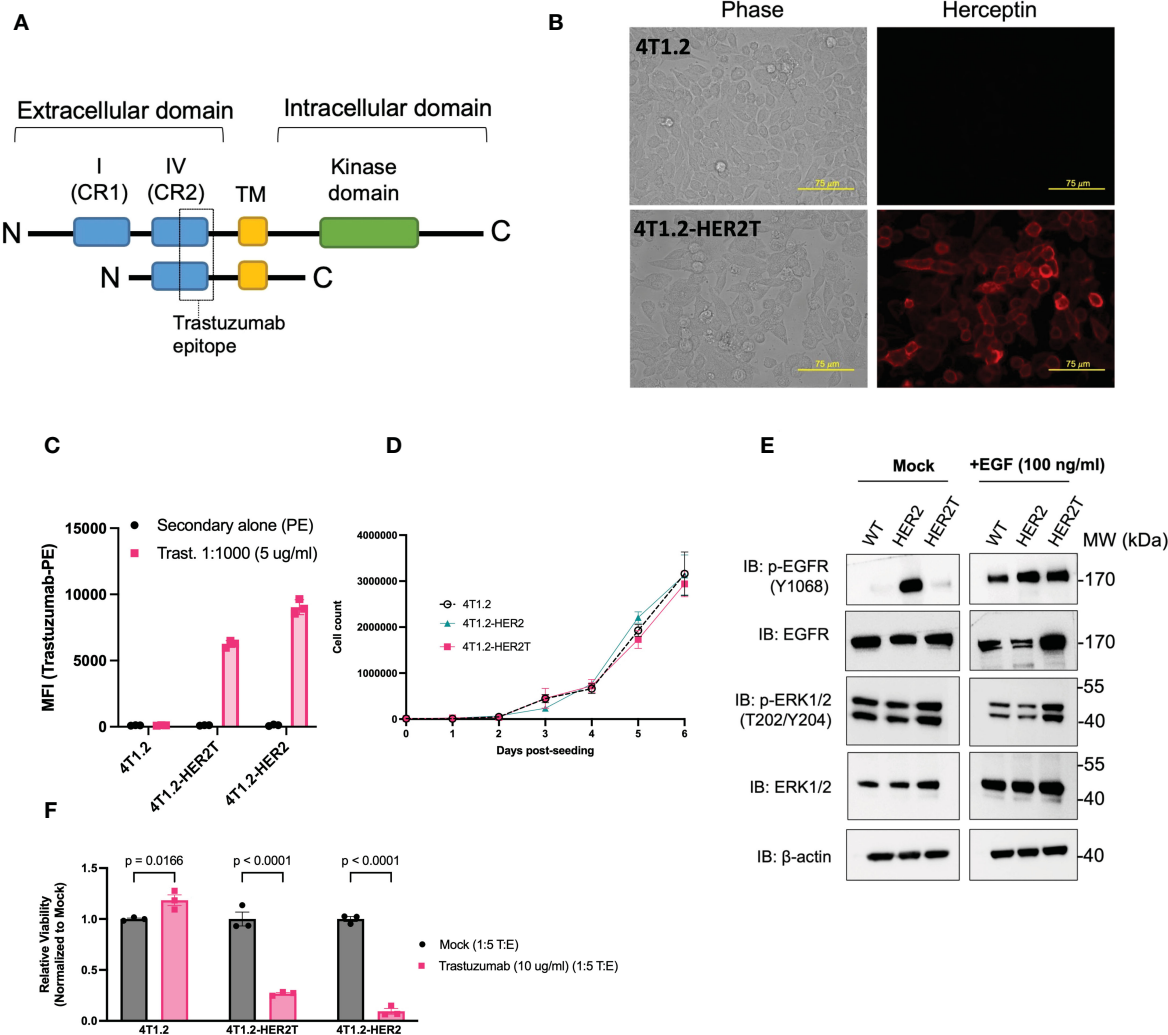
4T1.2 cells expressing HER2T bind to trastuzumab *in vitro.* **(A)** Diagrammatic representation of HER2T design **(B)** 4T1.2 and 4T1.2-HER2T cells were seeded on glass coverslips, fixed and stained with trastuzumab (1:1000), and visualized with Alexa Fluor 594-conjugated goat anti-human IgG secondary antibody. (20x magnification). **(C)** 4T1.2, 4T1.2-HER2T, and 4T1.2-HER2 cells were stained using trastuzumab (1:1000), followed by staining with PE-conjugated goat anti-human IgG secondary antibody. Geometric mean fluorescence intensity was obtained by flow cytometric analysis (geometric mean ± SEM; n = 3) **(D)** 7-day proliferation assay for 4T1.2, 4T1.2-HER2, 4T1.2-HER2T cells (mean ± SEM; n = 3) **(E)** Cells from **(D)** were plated and serum starved for 12 hours as indicated, followed by stimulation with EGF 100 ng/ml for 45 minutes as indicated. Whole cell lysates were extracted and resolved by PAGE and probed as shown. **(F)** Indicated cell lines were co-cultured with NK-92MI CD16a+ cells. Remaining target cells were stained with Coomassie blue, solubilized, and quantified at absorbance 595 nm (mean ± SEM; n=3; two-way ANOVA, Sidak’s correction for multiple comparison).

To generate an immunocompetent mouse model of huHER2-amplified cancer with improved tumour take rates and less spontaneous tumour regression, we engineered a truncated variant of HER2 (HER2T) from which the outermost immunogenic domains and intracellular kinase domain were removed (21) (**Figure 2A**). We preserved the extracellular cysteine rich domain IV containing the trastuzumab epitope, the transmembrane domain, and a short anchoring region of the C-terminus. This construct was stably expressed in 4T1.2 cells by lentivirus-mediated gene transfer and localized to the cell surface detected through immunofluorescence staining (**Figure 2B**) and flow cytometry (**Figure 2C**) using trastuzumab as the primary antibody. Cell surface expression levels were comparable to full length huHER2. Due to the truncated nature of HER2T, this construct lacks epitopes detectable by commercial Western immunoblotting antibodies, and may only be detected in non-denaturing conditions using trastuzumab.

To assess the potential impact of our HER2T construct on cell-intrinsic signal transduction, we confirmed that HER2T expression did not impact 4T1.2 cell proliferation, up to 7-days post-seeding (**Figure 2D**). Immunoblotting revealed that HER2T expression did not alter the phosphorylation patterns of epidermal growth factor receptor (EGFR) or ERK1/2, following stimulation of cells with 100 ng/ml EGF ligand or no ligand control (mock) in 4T1.2 (**Figure 2E**) and other cell lines (**Figures S1A, B**), as compared to full length HER2.

We next evaluated the potential for HER2T to engage NK cells and elicit antibody-dependent cell-mediated cytotoxicity (ADCC), a key mechanism of trastuzumab-mediated therapies (22–26). We co-cultured 4T1.2-HER2T cells with NK-92MI CD16a+ cells (**Figure S1 C**) in the presence of 10 μg/ml trastuzumab (T:E ratio 1:5) and observed significant ADCC (**Figures 2F**, **S1D**). The magnitude of NK-mediated target cell-killing was comparable between 4T1.2-HER2T and 4T1.2-HER2 cells, indicating the preserved epitope in HER2T is sufficient to induce functional binding of trastuzumab. Together, these results confirm the HER2T construct is expressed on the cell surface, and can bind to trastuzumab to elicit ADCC.

### 3.2 4T1.2-HER2T tumours exhibit improved take rate and reduced immunogenicity *in vivo*

**Figure 3:**
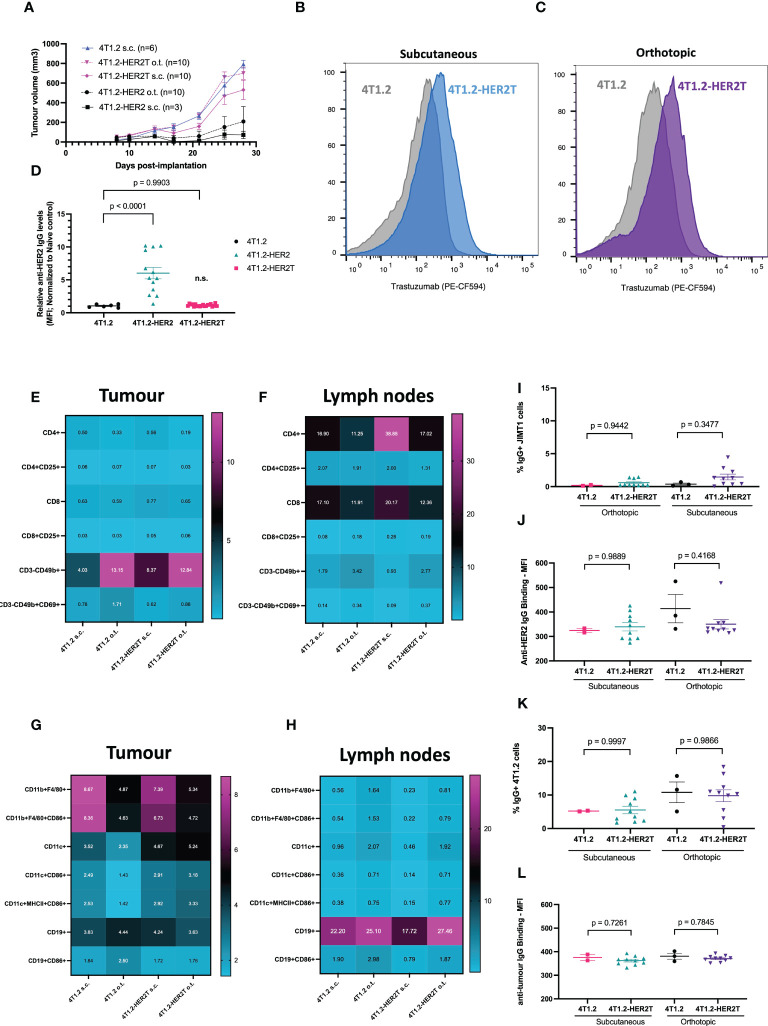
4T1.2-HER2T tumours exhibit improved take rate and reduced immunogenicity *in vivo.* **(A)** BALB/c mice were implanted with the indicated cells at 1 x 10^6^ seeding density, subcutaneously (s.c.) in the right flank, or orthotopically (o.t.) in the second mammary fat pad. Tumour progression was monitored through multiple weekly measurements with electronic calipers. **(B, C)** Mice were implanted with subcutaneous (blue, n=5) or orthotopic tumours (purple, n=5) as indicated. Tumours were harvested 25 days post-implantation, dissociated, and assessed for HER2T expression by flow cytometry.**(D)** Serum from 4T1.2 (n=6), 4T1.2-HER2 (n=13), 4T1.2-HER2T (n=20) tumour-bearing mice, or naïve controls, was collected 10 days post-implantation. Serum was incubated with JIMT1 for detection of HER2-reactive IgG by flow cytometry (mean ± SEM; one-way ANOVA with Dunnett’s correction for multiple comparison relative to 4T1.2) **(E–H)** BALB/c mice were implanted with 4T1.2 (s.c. n=2, o.t. n=3) or 4T1.2-HER2T (s.c. n=10, o.t. n=10) tumours. Tumours **(E, G)** and draining lymph nodes **(F, H)** were harvested 25 days post-implantation, processed, and analyzed by flow cytometry for immune characterization. **(I, J)** Serum from tumour-bearing mice from **(E–H)** was collected and assessed for HER2-reactive IgG at 25-days post-implantation. JIMT1 cells were incubated with serum, followed by anti-mouse IgG, and quantification by flow cytometry. **(K, L)** Serum from **(E–H)** was analyzed for anti-tumour serum by incubation of 4T1.2 cells with serum (mean ± SEM; n.s. not significant relative to 4T1.2 control; one-way ANOVA with Tukey’s correction for multiple comparison).

A polyclonal 4T1.2-HER2T-2 clone, herein referred to as “4T1.2-HER2T”, was selected for further studies given its mid-range expression levels (**Figure S1E**). We first assessed tumour take rates and baseline immunogenicity of 4T1.2-HER2T cells in BALB/c mice. We implanted 4T1.2, 4T1.2-HER2, and 4T1.2-HER2T at 1 x 10^6^ cells per mouse either subcutaneously (s.c.) or orthotopically in the mammary fat pad (o.t.) (**Figure 3A**). For subcutaneous tumours, take rates were 100% (6/6) for the 4T1.2 tumours, 33% (1/3) for the 4T1.2-HER2 tumours, and 90% (9/10) for the 4T1.2-HER2T tumours. For orthotopic mammary fat pad tumours implanted, take rates were 10% (1/10) for the 4T1.2-HER2 tumours, and 100% (10/10) for the 4T1.2-HER2T tumours.

**Figure 4:**
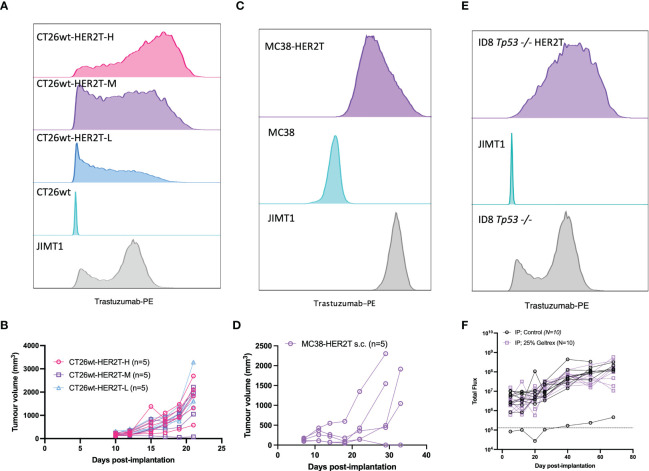
HER2T expression does not compromise tumourigenesis in common syngeneic tumour models. **(A)** CT26wt-HER2T clones expressing different levels of HER2T were assessed by flow cytometry. **(B)** CT26wt-HER2T Cells were implanted s.c. in BALB/c mice (5 x 10^5^ cells + Geltrex) in the right flank and monitored for tumour progression (n=5 mice per clone). **(C)** MC38 and MC38-HER2T cells were assessed for HER2T levels by flow cytometry. **(D)** MC38-HER2T cells were implanted s.c. (2 x 10^6^ cells + Geltrex) in C57BL/6 mice (n=5) and monitored for tumour progression. **(E)** ID8 *Tp53-/-* HER2T Fluc, or control, cells were assessed for HER2T levels by flow cytometry. **(F)** Cells were implanted i.p.,with (n=10) or without (n=10) Geltrex, in C57BL/6 mice. Luminescence was measured by weekly IVIS imaging; the dotted line represents background luminescence.

To confirm HER2T expression within tumours, BALB/c mice were implanted with 4T1.2 and 4T1.2-HER2T tumours either subcutaneously or orthotopically. Tumours were extracted D25 post-implantation, dissociated, and HER2T levels were quantified by flow cytometry or qRT-PCR, revealing retention of cell surface HER2T (**Figures 3B, C**) and HER2T mRNA expression (**Figure S2A**). Syngeneic models, derived from other cell lines transduced with HER2T, also led to >80% tumour take rates. These models include CT26wt murine colorectal subcutaneous tumours (15/15, 100%) (**Figures 4A, B**), MC38 murine colorectal subcutaneous tumours (4/5, 80%) (**Figures 4C, D**), and ID8 *Tp53* -/- murine high-grade serous carcinoma intraperitoneal tumours (19/20, 95%) (**Figures 4E, F**).

To characterize naturally arising anti-tumour immune responses upon 4T1.2-HER2T tumour implantation, serum anti-HER2T IgG (**Figure 3D**) was measured at D10 post-implantation, revealing minimal HER2T-reactive IgG in 4T1.2 or 4T1.2-HER2T tumour bearing mice, contrary to higher levels observed in 4T1.2-HER2 tumour bearing mice. We next aimed to compare the status of immune populations within both the 4T1.2 parental and 4T1.2-HER2T tumour models. We implanted 4T1.2 or 4T1.2-HER2T tumours in BALB/c mice subcutaneously or orthotopically. D25 post-implantation tumours and tumour draining lymph nodes (TdLN) were harvested, dissociated, and interrogated by flow cytometry for T-cell and NK cell populations (**Figures 3E, F**, **S2B**) as well as B-cell and other antigen presenting cell populations (**Figures 3G, H**, **S2C**). We observed consistency in immune population distribution and activation status between 4T1.2 and 4T1.2-HER2T tumours and TdLN, except for increased CD4+ T-cells within the subcutaneous 4T1.2-HER2T TdLN. This increase was approximately 2-fold of the CD4+ population found in parental tumours. Broader significant differences were noted between subcutaneous and orthotopic tumours rather than between 4T1.2-HER2T and parental 4T1.2 tumours (**Figures S2D, E**). Specifically, we observed increased proportions of CD11b+F4/80+ macrophages, and decreased proportions of CD3-CD49b+ NK cells within subcutaneous tumours, compared to orthotopic tumours.

We observed comparably low levels of HER2-reactive serum IgG between the HER2T and parental tumours at D25 post-implantation (**Figures 3I, J**), consistent with our observations at D10 (**Figure 3D**). There were no differences in serum IgG recognizing 4T1.2-associated antigens between 4T1.2-HER2T and parental 4T1.2 groups (**Figures 3K, L**). To determine whether low levels of anti-HER2 serum IgG could elicit ADCC, we co-cultured serum-treated HER2+ JIMT1 with CD16a+ NK-92MI cells (**Figure S2F**). We detected no observable ADCC in any serum-treated conditions, all of which were comparable to naïve mouse serum, and significantly lower than the trastuzumab positive control. Similarly, following culture of JIMT1 cells with mock or heat-inactivated serum for 24 hours, we detected no impact on cell viability thereby excluding a role for complement-mediated killing (**Figure S2G**). Serum cytokine and chemokine levels in 4T1.2 tumour-bearing mice were also similar irrespective of HER2T expression, but distinct from control MC38 tumour bearing mice (**Figure S2H**). Using IFN-γ ELISpot performed on peripheral blood lymphocytes isolated from 4T1.2 or 4T1.2-HER2T tumour-bearing mice, each stimulated with either 4T1.2 or 4T1.2-HER2 lysates, no appreciable differences in overall antigenic response were observed (**Figure S2I**).

We moreover observed similar disease progression between 4T1.2 and 4T1.2-HER2T tumours, and similar tumour histology (**Figure S2J**), the latter of which was assessed by a pathologist. Consistent with the observation of their similar progression characteristics, these results suggest 4T1.2 and 4T1.2-HER2T tumour models are nearly equivalent in terms of baseline cytokine profiles, humoral and cellular anti-tumour responses.

### 3.3 4T1.2 cells expressing HER2T respond to VSVΔ51+T-DM1 combination *in vitro*

**Figure 5:**
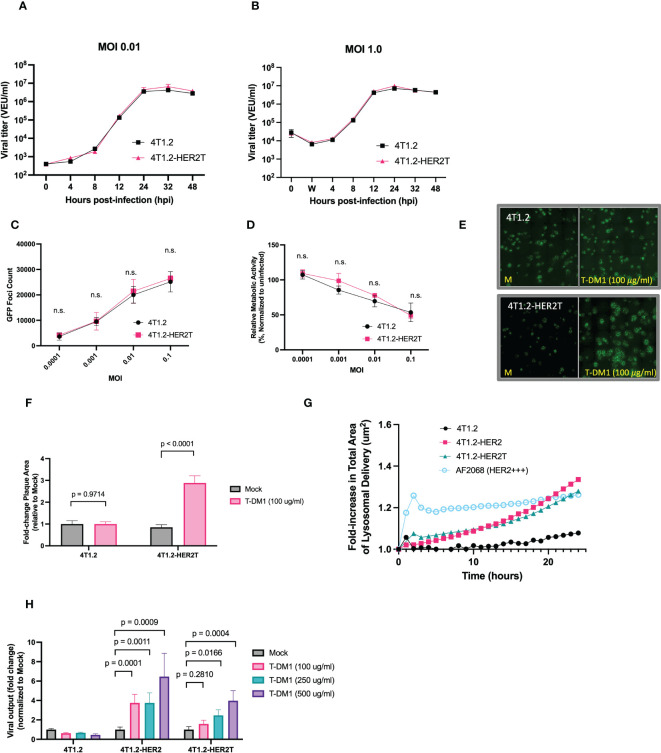
4T1.2-HER2T cells are sensitized to VSVΔ51 infection following treatment with T-DM1. **(A, B)** 4T1.2 or 4T1.2-HER2T cells were infected at **(A)** low or **(B)** high MOI with VSVΔ51-Fluc. Viral supernatant was collected at the indicated timepoints and viral titer was quantified. **(C, D)** Cells were infected with different MOI of VSVΔ51-GFP. **(C)** GFP foci were counted at 24 hpi, and **(D)** viability of infected cells was assessed by AlamarBlue at 48 hpi (n=3 per MOI; mean ± SEM; n.s. by multiple unpaired two-tailed t-tests). **(E)** Plaque expansion assay of the indicated cells. Monolayers were treated as indicated then infected with VSVΔ51-GFP at MOI 0.001 for 45 minutes. Viral supernatant was removed and a 0.5% agarose-DMEM overlay was added. Cells were incubated for 48 hours then imaged. **(F)** Plaque diameters from **(E)** were quantified using ImageJ and fold change calculated (mean ± SEM; n=3 wells, 40 plaques quantified randomly per well, two-way ANOVA, Sidak’s correction for multiple comparison) **(G)** trastuzumab internalization assay; cells were incubated with a pH-sensitive internalization reagent mixed with trastuzumab, incubated for 24 hours, and imaged at 30-minute intervals. Pixel intensity was quantified. **(H)** The indicated cell lines were treated as shown, then infected with MOI 0.01 VSVΔ51-Fluc. Virus titer was quantified 48 hpi (mean ± SEM; n=3 wells; two-way ANOVA with Dunnett’s correction for multiple comparisons).

To validate the suitability of the 4T1.2-HER2T model for assessment of a VSVΔ51-based therapy, we first verified that this cell line exhibits similar VSVΔ51 infectability to 4T1.2 parental cells. VSVΔ51 growth kinetics were assessed at low and high multiplicity of infection (MOI) (**Figures 5A, B**) and were determined to be comparable to the parental 4T1.2 cell line. We confirmed that 4T1.2-HER2T cells infected at different MOI of VSVΔ51-GFP exhibited similar viability and GFP transgene expression as 4T1.2 cells at 24-48 hours post-infection (hpi) (**Figures 5C, D**).

Through viral plaque expansion assay, we demonstrate a ~2.5-fold increase in viral plaque diameters following pre-treatment with 100 μg/ml T-DM1 in 4T1.2-HER2T cells, with no impact of T-DM1 on plaques formed on parental 4T1.2 (**Figures 5E, F**). We also demonstrated that trastuzumab internalization by 4T1.2-HER2T cells was comparable to 4T1.2-HER2, as this mechanism is critical for the release of the T-DM1 payload (**Figure 5G**).

Similarly, treatment with increasing concentrations of T-DM1 and infection with MOI 0.01 of VSVΔ51-Fluc led to a significant enhancement in viral output 48 hpi from 4T1.2-HER2 and 4T1.2-HER2T cells in a dose-responsive fashion, with no viral enhancement observed with T-DM1 in parental 4T1.2 cells (**Figure 5H**). Altogether these data suggest that 4T1.2-HER2T cells can efficiently internalize T-DM1 and retain susceptibility to VSVΔ51 comparable to parental 4T1.2 cells *in vitro*. 4T1.2-HER2T cells also demonstrate increased viral output following treatment with the combination of VSVΔ51+T-DM1 to the same extent as 4T1.2-HER2.

### 3.4 Treatment of 4T1.2-HER2T tumours with VSVΔ51+T-DM1 combination leads to durable cures and reduced lung metastasis *in vivo*

**Figure 6:**
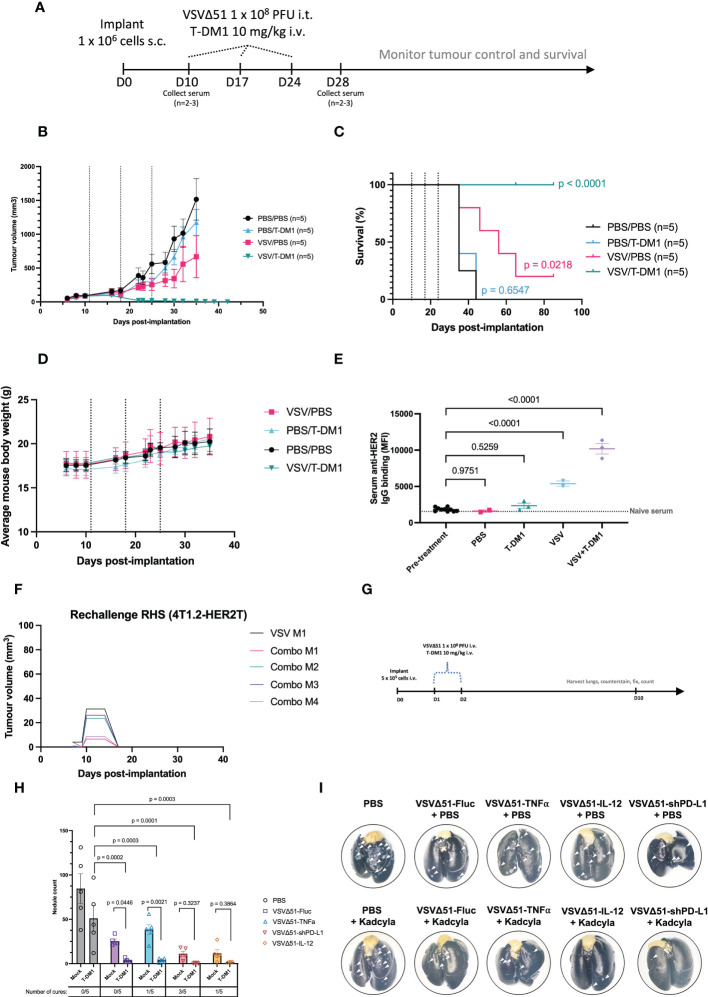
Treatment of 4T1.2-HER2T tumours with VSVΔ51 and T-DM1 combination leads to durable cures and reduced lung metastasis *in vivo.* **(A)** Tumour implantation and treatment regimen timeline **(B, C)** Following implantation and treatment as in **(A)**, mouse **(B)** tumour control and **(C)** survival were monitored. Dotted lines indicate treatments. Survival data p-values relative to PBS control. **(D)** Mouse weights measured over time as a wellness indicator. **(E)** Serum was collected from mice 4 weeks post-treatment. HER2-reactive IgG was measured by incubation with JIMT1 cells. The dotted line represents naïve mouse serum control. (mean ± SEM, PBS n=2, T-DM1 n=3, VSVΔ51 n=2, VSVΔ51+T-DM1 n=3; one-way ANOVA with Dunnett’s correction for multiple comparisons, relative to pre-treatment) **(F)** Cured mice from **(A–C)** were rechallenged with 4T1.2-HER2T cells (1 x 10^6^ cells) s.c. in the contralateral flank. **(G)** Tumour implantation and treatment regimen timeline for the i.v. lung metastases model **(H)** Following harvest of lungs, staining, and fixation, metastatic nodules were counted (mean ± SEM, n=5; p-value indicated relative to T-DM1 alone (two-way ANOVA with Dunnett’s correction for multiple comparison), or relative to individual VSVΔ51-alone conditions (two-way ANOVA with Fisher’s LSD test). Tumour-free mice indicated as “cures”. **(I)** Representative images of lungs post-harvest. White arrows indicate surface nodules.

To assess the responsiveness of the 4T1.2-HER2T tumour model to treatment with VSVΔ51+T-DM1, we treated (13, 14) subcutaneous 4T1.2-HER2T tumours as outlined (**Figures 6A–D**, **S3A**). Following treatments, we observed a steady continuous increase in mouse weights and no changes in mouse wellness, suggesting that the treatment regimen was well-tolerated (**Figure 6D**). Subcutaneous 4T1.2-HER2T tumours progressed rapidly in PBS treated control groups with a median overall survival (mOS) of 35 days (**Figure 6C**). Combination treatment with VSVΔ51+T-DM1 led to complete tumour regression in 100% of the mice (5/5) (**Figures 6B, C**). VSVΔ51 monotherapy led to significant stunting in tumour progression and prolonging of mOS to 56 days, and complete tumour regression in 20% of mice (1/5). T-DM1 monotherapy led to modest stunting in tumour growth, with no impact on mOS compared to PBS control. Interestingly, we observed a significant increase in anti-HER2 serum IgG 4 weeks post-treatment in VSVΔ51+T-DM1 combination treated mice compared to monotherapy, PBS, or pre-treatment baseline serum IgG levels (**Figure 6E**). All cured mice were subsequently subjected to tumour rechallenge at D90 following initial implantation, and demonstrated complete rejection of the 4T1.2-HER2T rechallenge (**Figure 6F**).

Substituting trastuzumab control for T-DM1 at the same dose abolished the curative effects of the combination with VSVΔ51 (**Figures S3B, C**) in the 4T1.2-HER2T model. Treatment with trastuzumab alone yielded a modest improvement (mOS 55 days) compared with PBS control (mOS 46 days), and although the combination of trastuzumab+VSVΔ51 (mOS 81 days) performed better than monotherapies, it was substantially less effective than the combination of T-DM1+VSVΔ51 (100% survival). Furthermore, treatment of parental 4T1.2 tumours with T-DM1, whether alone or in combination with VSVΔ51, had no impact on tumour progression or overall survival (**Figures S3D, E**). However, treatment of 4T1.2 tumours with VSVΔ51 yielded a modest improvement in tumour control (mOS 34-38 days) compared to PBS control (mOS 31 days). These findings highlight the curative potential of T-DM1+VSVΔ51 in an immunocompetent mouse setting, and moreover underscore the DM1 conjugate as a requisite for the mechanistic synergy observed with VSVΔ51.

Given the robust response observed in this subcutaneous tumour model, we next aimed to assess the potency of the combined OV/ADC therapeutic strategy in an advanced disease stage model. For these studies, we injected 4T1.2-HER2T tumours intravenously and used the treatment regimen outlined in **Figure 6G**. Quantification of metastatic lung nodules indicated a significant reduction following VSVΔ51-Fluc+T-DM1 treatment (3.4 nodules), compared to VSVΔ51 (25.4 nodules) or T-DM1 (51 nodules) monotherapy, or PBS control (84.6 nodules) (**Figures 6G–I**). We observed enhanced efficacy of VSVΔ51+T-DM1 and complete cures when mice were treated with VSVΔ51 variants encoding immune-modulatory payloads. These payloads include TNFα (27) (1/5 cures, 3.8 nodules), shPD-L1 (28) (3/5 cures, 0.6 nodules), or interleukin-12 (29, 30) (IL-12) (1/5 cures, 1.75 nodules). Taken together, our data indicate VSVΔ51+T-DM1 yields mechanistic synergy and subsequent therapeutic efficacy in both early-stage and late-stage disease models in a HER2T-dependent fashion. Moreover, the efficacy of this approach is amenable to improvement through modification of the OV with additional payloads.

### 3.5 Treatment combination of VSVΔ51+T-DM1 leads to humoral anti-tumour response and immunologic memory

**Figure 7:**
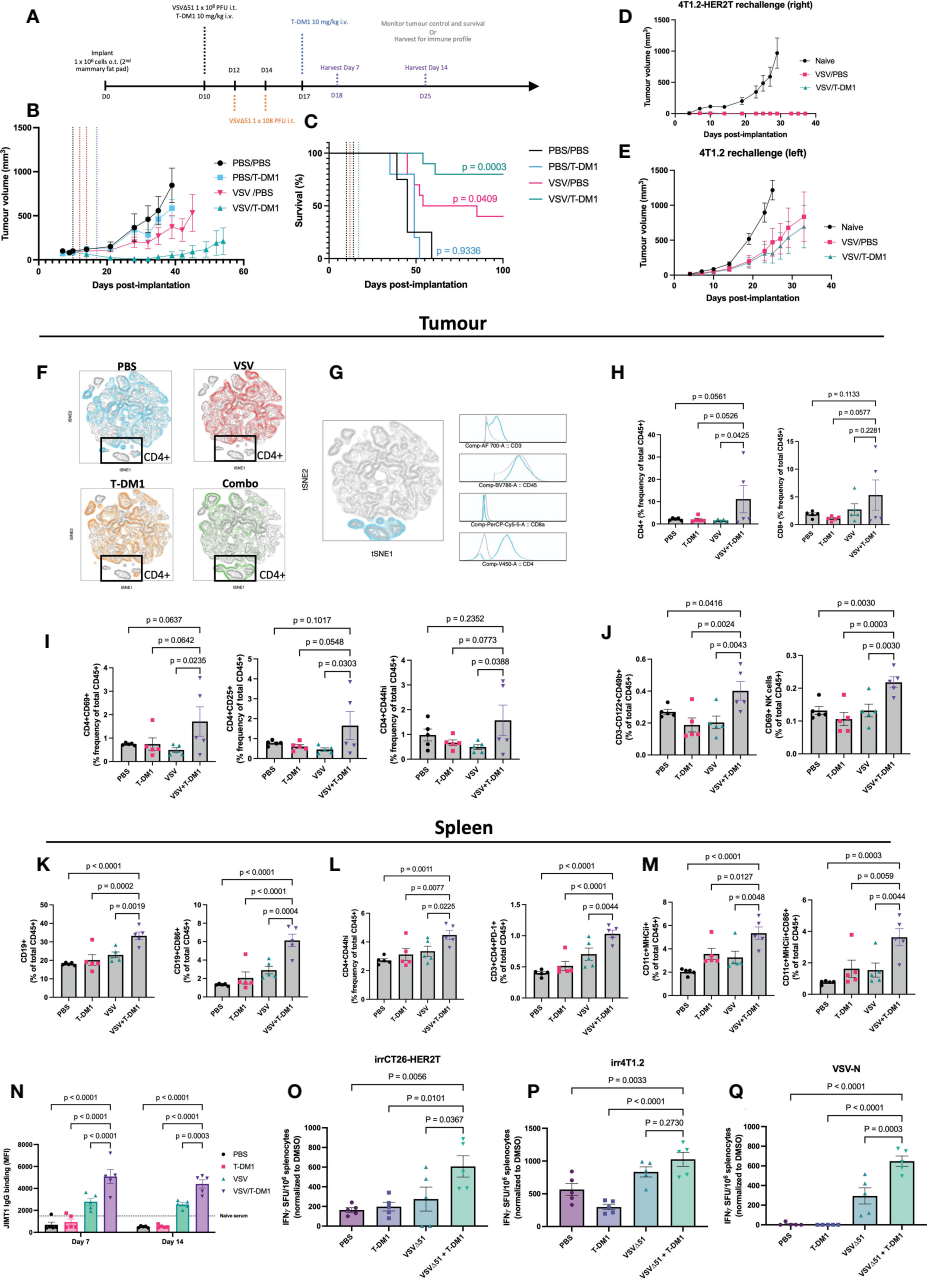
Treatment combination of VSVΔ51 + T-DM1 leads to humoral anti-tumour response and immunologic memory. **(A)** Implantation of 4T1.2-HER2T o.t. tumours and treatment regimen timeline **(B, C)** BALB/c mice were implanted with 4T1.2-HER2T o.t. tumours and treated as indicated (n=5 per group). Tumour volumes **(B)** and survival **(C)** were monitored. Survival data p-values relative to PBS control. **(D, E)** Cured mice from **(B, C)** were rechallenged with **(D)** 4T1.2-HER2T tumours in the right side or **(E)** 4T1.2 parental tumours in the left side, of the 3^rd^ mammary fat pad. **(F–M)** Following treatment, n=5 mice were harvested per group at day 7 post-treatment. **(F)** opt-SNE and FIt-SNE algorithms applied to D7 T-cell data. Overlays for each treatment group is indicated. **(G)** Identification of CD4+ signature in the highlighted (blue) population using multigraph overlays. **(H)** Tumour-infiltration total CD4+ and CD8+ T-cell populations **(I)** tumour-infiltrating activated CD4+ T-cells (CD69, CD25, CD44hi), **(J)** Tumour total and activated CD69+ NK cells, **(K)** Splenic total and activated CD86+ B-cell, **(L)** Splenic activated CD4+ T-cells (CD44hi or PD-1+), **(M)** Splenic total and activated CD86+ dendritic cells, **(N)** Serum was collected from mice at D7 and D14, and assessed for HER2-reactive IgG. (For all bar charts: mean ± SEM, n=5 per group; p-value calculated by one-way ANOVA, or two-way ANOVA for **(L)**, with Fisher’s LSD test). Representative contour plots shown in **Figure S5**. **(O–Q)** IFNγ ELISPOT results from murine splenocytes isolated from the spleens at D14, and stimulated with **(O)** irradiated CT26-HER2T cells, **(P)** irradiated 4T1.2 cells, or **(Q)** VSV-N peptide. (mean ± SEM, n=5 per group; p-value calculated by one-way ANOVA, with Dunnett’s correction for multiple comparisons).

We next evaluated the VSVΔ51+T-DM1 combination strategy using the orthotopic mammary fat pad implantation model. For this model, we condensed our treatment regimen into a 7-day schedule (**Figure 7A**) as opposed to the standard 21-day schedule. A condensed schedule would support the harvest of tissues at D7 and D14 following the first treatment, for the analysis of anti-tumour immune responses. To do this, we administered three doses of VSVΔ51 intratumourally one day apart. Two intravenous doses of T-DM1 were administered: one with the first VSVΔ51 injection, and the second dose 7 days after. The rationale for spacing T-DM1 doses one week apart is because this ADC has a 14-21 day half-life.

This treatment regimen led to consistent stunting of tumour progression and 80% overall survival (8/10) in the VSVΔ51+T-DM1 combination group. In comparison, VSVΔ51 monotherapy yielded 40% (4/10) overall survival and a 69.5 mOS, while T-DM1 monotherapy yielded 0% survival but modest improvement in mOS to 49 days, from 45 days with PBS control (**Figures 7B, C**, **S4A**).

All cured mice were rechallenged at D90 post-initial implantation with bilateral tumours: 4T1.2 parental (lefthand side) and 4T1.2-HER2T (righthand side). Monitoring of tumour progression revealed that all HER2T-expressing tumours were rejected in all mice compared to naïve controls (**Figures 7D**, and **S4B**). Furthermore, 4T1.2 tumour challenges led to rejection in 3/7 (43%) of the VSVΔ51+T-DM1 cured mice, and 2/5 (40%) of the VSVΔ51 cured mice (**Figures 7E**, **S4C**). Despite rejection of only ~40% of the non-HER2T-expressing parental tumour rechallenges, the progression of all 4T1.2 tumours was noticeably stunted in all cured mice.

To further characterize the immune response following treatment in the orthotopic 4T1.2-HER2T tumour model, mice were euthanized at D7 and D14 following the first treatment dose (**Figure 7A**). Sera were collected for IgG testing, as well as tumours, TdLN, and spleens to measure cell-mediated immune responses (**Figures S5A–D**). Our analyses revealed robust responses at D7, including tumour immune infiltration by activated lymphocytes, much of which had dissipated by D14. As an overall initial analysis of immune populations, mouse tumour T-cell data from D7 were first annotated and concatenated. T-distributed stochastic neighbor embedding (tSNE) plots were generated, which were used to identify clustering patterns (**Figure 7F**). Unique clusters in the VSVΔ51+T-DM1 “Combo” treatment group were traced back to CD4+ T-cell signatures (**Figure 7G**). Upon examination of the CD3+ population, a significant enrichment of CD4+ and CD8+ T-cells within the tumour compartment at D7 was observed (**Figure 7H**, **S5E**) in mice that had received VSVΔ51+T-DM1, compared to those that received monotherapies or PBS control. Although we observed no impact of VSVΔ51+T-DM1 on the activation status of CD8+ T-cells in tumours, TdLN, or spleens (**Figure S4D**), our analyses revealed an average 2-fold increase in activated CD44hi, CD25+, or CD69+ tumour-infiltrating CD4+ T-cells in the same treatment group (**Figures 7I**, **S5F, G**), relative to PBS or monotherapies. We also observed a trend toward reduced T_reg_ populations within the TdLN of VSVΔ51+T-DM1 treated mice, compared to monotherapies (-27%) or PBS (-37%) controls (**Figures S4E, F**).

Interrogation of the NK cell population revealed a significant 1.5-2-fold increase in total CD3-CD122+CD49b+ NK cells within the tumour compartment of VSVΔ51+T-DM1 treated mice compared to monotherapies or PBS controls (**Figures 7J**, and **S5H, I**) at D7. We moreover observed a similar increase in early activated CD69+ NK cells in the combination treatment groups, suggesting greater tumour infiltration by activated NK cells. This increase in NK cell infiltration was coupled with a modest increase in the total levels of MHCI in CD45- tumour cells at both D7 and D14 post-treatment (**Figures S4G, H**).

To look at systemic anti-tumour responses, we examined the immune profiles of the spleens of all treated mice. Most prominently, we identified a significant 10-12% enhancement in the total levels of CD19+ splenic B-cells in VSVΔ51+T-DM1 treated mice compared to monotherapies or PBS control, coupled with a significant 2-4 fold increase in the levels of CD86+ activated B-cells (**Figures 7K**, **S5J**). Interestingly we also noted a consistent 1.5-2-fold increase in activated PD-1+ or CD44hi CD4+ T-cells (**Figure 7L**) in the combination-treated mice. Total CD11c+MHCII+ dendritic cells (DCs) exhibited a 2-3-fold increase, while activated CD86+ DCs exhibited a 2-4-fold increase (**Figures 7M**, **S5K, L**), in the spleens of VSVΔ51+T-DM1 treated mice, compared to monotherapies or PBS controls.

Following assessment of anti-HER2 serum IgG by flow cytometry on HER2+ JIMT1 cells at both D7 and D14, we detected pronounced levels of anti-HER2 IgG especially in VSVΔ51 or VSVΔ51+T-DM1 treated mice. Despite a high percentage of IgG+ JIMT1 cells in the VSVΔ51 groups (**Figure S4I**), the total staining intensity was significantly greater (~2-fold) in the anti-HER2 IgG repertoire of the VSVΔ51+T-DM1 groups (**Figure 7N**). In the T-DM1 monotherapy and PBS control groups, IgG staining intensity was very low, and comparable to naïve serum non-specific binding.

We furthermore assessed the spleens of mice harvested at D14 for antigen-specific responses by ELISpot (**Figures 7O–Q**, **S4J**). We report that mice from the VSVΔ51+T-DM1 group demonstrated a significant increase in IFN-γ secreting cells in response to stimulation by irradiated CT26-HER2T cells, highlighting strong HER2T-specific responses following treatment (**Figure 7O**). We moreover observed a robust response to VSV-N peptide stimulation in the VSVΔ51+T-DM1 treatment group, significantly greater than in the VSVΔ51 monotherapy group, despite administration of identical doses of VSVΔ51 (**Figure 7P**). We also observed an increase in the number of IFN-γ secreting cells in response to stimulation by irradiated 4T1.2 cells, suggesting treatment also induces a broadly anti-tumour immune response (**Figure 7Q**).

Taken together, these findings suggest that combination treatment with VSVΔ51+T-DM1 incites T-cell and NK tumour infiltration, followed by stimulation of a systemic anti-tumour response. In addition, these data implicate a CD4-B-NK axis with the generation of and the response to anti-tumour humoral immunity through IgG targeted to the tumour-specific HER2T antigen.

## 3 Discussion

While it cannot substitute for human trials, we believe the 4T1.2-HER2T model developed in this study has characteristics of high-grade “HER2+” mBC characteristic of Stage IV human mBC (31, 32), and can be a useful tool to complement and overcome many limitations of existing transgenic and xenograft models.

Expression of full-length HER2 in 4T1.2 cells is highly immunogenic and is documented to induce spontaneous tumour regression in wildtype mice, as confirmed here (6–8, 33) (**Figures 1A, B, F**). While full-length HER2 has been used to establish some syngeneic tumour models, these models are typically non-metastatic or of non-mammary origin (7, 34–37), therefore being unrepresentative of high-grade mBC. These models have afforded utility in assessing anti-HER2 vaccines (38, 39) or anti-neoplastic agents (40–42). For this reason, we engineered a truncated version of HER2, HER2T, that lacks highly immunogenic ectodomains (7), and has no kinase activity (**Figures 2A, D, E**) to avoid altering intracellular signaling pathways. The trastuzumab epitope was retained, allowing for the evaluation of many therapeutic strategies that target this epitope, including the OV/ADC combination strategies tested in this study.

We demonstrate here that the minimal HER2T epitope can both bind trastuzumab and elicit ADCC (**Figure 2F**), a key mechanism of trastuzumab efficacy (23, 24, 26). We moreover show that the HER2T construct is minimally immunogenic in 4T1.2 tumours (**Figure 3D**) and can be expressed at different levels (**Figure S1E**), which is useful given HER2-low cancers have been recently reclassified as relevant for HER2-targeted therapy (43). The ability to express HER2T in multiple cell lines for the generation of different HER2T+ syngeneic tumour models is also advantageous (**Figure 4**). For example, we have generated such a model using ID8 *Tp53* -/- providing a model of murine HER2+ ovarian cancer, which to our knowledge did not previously exist despite frequent HER2 overexpression in this subtype (44, 45).

In addition to successful implantation of 4T1.2-HER2T tumours in wildtype BALB/c mice, both subcutaneously and orthotopically (**Figure 3A**), we confirmed retention of HER2T expression up to at least D25 post-implantation (**Figures 3B, C**, and **S2A**). Profiling of the tumour immune microenvironment revealed similar abundancies of different immune populations at D25 post-implantation between 4T1.2 and 4T1.2-HER2T tumours (**Figures 3E–H**). We observed noticeable differences in the lymphoid composition of the tumour immune microenvironment and TdLN depending on subcutaneous *vs.* orthotopic implantation, regardless of HER2T expression.

Strikingly, VSVΔ51+T-DM1 combination therapy led to a 100% survival rate using the subcutaneous 4T1.2-HER2T model (**Figures 6A–C**), and potent immunologic memory (**Figure 6F**) in a HER2-preferential fashion (**Figures S3D, E**). Similarly high but incomplete cure rates (80% survival) were observed using a compressed regimen in orthotopic tumours (**Figures 5B–E**). While we observed very high effectiveness of the VSVΔ51+T-DM1 combination in the lung metastases model based on lung nodule counts (**Figures 6H, I**), this did not lead to cures without incorporation of an immune-enhancing transgene. In this regard, best results were achieved using an shPD-L1-encoding virus (28) from a cassette that enables exosome packaging and action at a distance (3/5 mice cured).

Following early observations of enhanced anti-HER2 serum IgG following treatment with VSVΔ51+T-DM1 (**Figure 6E**), we sought to inspect the CD4-B-NK axis as a potential effector. We reported significant elevation in the level of CD4+ and CD8+ T-cell tumour infiltration (**Figure 7H**), with increased CD4+ activation status (**Figure 7I**) at D7 post-treatment. We also noted significantly enhanced levels of NK cell infiltration and activation (**Figure 7J**) within tumours. Spleens of mice receiving combination treatment had higher levels of total and activated B-cells (**Figure 7K**), effector CD44hi or PD-1+ CD4+ T-cells (**Figure 7L**), and total and activated dendritic cells (**Figure 7M**). Together with the high levels of HER2-reactive serum IgG levels (**Figure 7N**), our data suggest that VSVΔ51+T-DM1 treatment elicits a humoral response centered on CD4+ T-cell tumour infiltration and activation, systemic dendritic cell activation by VSVΔ51 or T-DM1, followed by anti-tumour IgG production. These findings are consistent with clinical observations documenting a correlation between the presence of elevated anti-HER2 autoantibodies and improved outcome (46). This cascade is targeted toward a tumour-specific antigen, for which previously established tolerance has now been broken. Similar observations have been previously made in OV studies (47), underscoring the fundamentally immune-stimulatory nature of OVs.

Notably, challenging mice cured of 4T1.2-HERT tumours with parental 4T1.2 cells (**Figure 7E**) led to a 40% tumour rejection rate, and tumour growth was stunted. This observation implicates antigen-spread toward the parental 4T1.2 tumour antigens. Indeed, we had identified broadly tumour-reactive cell-mediated systemic responses in VSVΔ51+T-DM1 treated mice, against both HER2T and parental 4T1.2 cells (**Figures 7O, P**). HER2 downregulation, epitope mutation, or signaling aberrations often render tumours resistant to trastuzumab-based therapeutics in the clinic (48). Immunotherapies involving pro-inflammatory oncolytic agents such as VSVΔ51, are thought to expose tumour antigens that help augment the anti-tumour immune response. This is a key feature that can help prevent tumour escape through mechanisms that downregulate the originally-targeted tumour-associated antigen, such as HER2 explored here (49, 50). This effect can be further enhanced through the upregulation of MHCI in tumours (49). Indeed, we observed a modest increase in MHCI in all treatment groups at D14 (**Figure S4H**).

In conclusion, the syngeneic model simulating human HER2+ murine breast cancer we have developed underscores the efficacy of VSVΔ51+T-DM1 combination therapy regimens in an immunocompetent setting. This efficacy correlates with robust CD4-DC-B-NK immune signatures, as well as increased anti-HER2 serum IgG. Our findings therefore suggest a role for humoral immunity in the anti-tumour response following VSVΔ51+T-DM1 treatment. Nevertheless, one limitation of the HER2T model is that it does not capture potential off-target toxicities, since HER2T is only expressed in the tumour. This aspect may be addressed using other models (5, 51) capable of accounting for certain HER2-targeted toxicities (3, 52). Moreover, with the design of HER2T intended for simulation of trastuzumab-reactive “HER2-positive” status with no kinase domain, this model is not compatible with the evaluation of kinase inhibitors or biologics targeted against a non-trastuzumab epitope, such as pertuzumab. However, HER2T may be expressed across a range of different murine lines without impacting tumourigenicity, highlighting the utility of the HER2T syngeneic model as a practical and accessible tool for assessment of other anti-HER2 therapeutics utilizing the trastuzumab paratope, which include ADCs, antibodies, CAR-T, and T-cell engagers (19).

## Data availability statement

The raw data supporting the conclusions of this article will be made available by the authors, without undue reservation.

## Ethics statement

*In vivo* experiments were performed *via* protocols OHRI-2265 and OHRI-2264 which are in good standing with the Animal Care Committee, and care and treatment of animals was in accordance with the ethical standards of the Canadian Council on Animal Care and with the Animals for Research Act.

## Author contributions

ZT, RA, and J-SD participated in conceptualization and methodology design of the studies. J-SD and RA, provided supervision, resources, guidance, and funding acquisition for the project. ZT, MC, KN, and JS performed *in vitro* investigation. ZT, MC, NA, AC, MT, and MS performed and assisted with *in vivo* animal investigation. ZT, MC, and NA performed flow cytometry preparation and acquisition. ZT and FF performed formal analyses. FF provided histologic assessment of tumour specimens. ZT performed visualization, and project administration. ZT, MC, KN, JS, and EL performed validation experimentation. ZT, MC, NA, and AC propagated, purified, and validated VSVΔ51. ZT prepared the original manuscript with editorial contributions from RA, JS-D, MC, NA, CI, and JB. All authors contributed to the article and approved the submitted version.
