# Supplementary material for: Syngeneic mouse model of human HER2+ metastatic breast cancer for the evaluation of trastuzumab emtansine combined with oncolytic rhabdovirus
Source: Front Immunol. 2023 Apr 19;14:1181014. doi: 10.3389/fimmu.2023.1181014 (PMC10154558; doi:10.3389/fimmu.2023.1181014)
Supplement: Supplementary file 1 [file DataSheet_1.pdf]

Supplementary Material

Syngeneic mouse model of human HER2+ metastatic breast cancer for the evaluation of trastuzumab emtansine combined with oncolytic rhabdovirus

Zaid Taha[1,2], Mathieu J.F. Crupi[1,2], Nouf Alluqmani[1,2], Faiha Fareez[3], Kristy Ng[1], Judy Sobh[1], Emily Lee[1], Andrew Chen[1], Max Thomson[1], Marcus M. Spinelli[1], Carolina S. Ilkow[1,2], John C. Bell[1,2], Rozanne Arulanandam[1,†], Jean-Simon Diallo[1,2,†,*]

[1] Centre for Cancer Therapeutics, Ottawa Hospital Research Institute, Ottawa, Ontario, K1H 8L6, Canada.

[2] Department of Biochemistry, Microbiology, and Immunology, Faculty of Medicine, University of Ottawa, Ottawa, Ontario, K1H 8M5, Canada.

[3]Department of Pathology and Molecular Medicine, McMaster University, Hamilton, Ontario, L8S 4L8, Canada.

[†] Contributed equally and share senior authorship

***Correspondence** should be addressed to Jean-Simon Diallo (jsdiallo@ohri.ca)

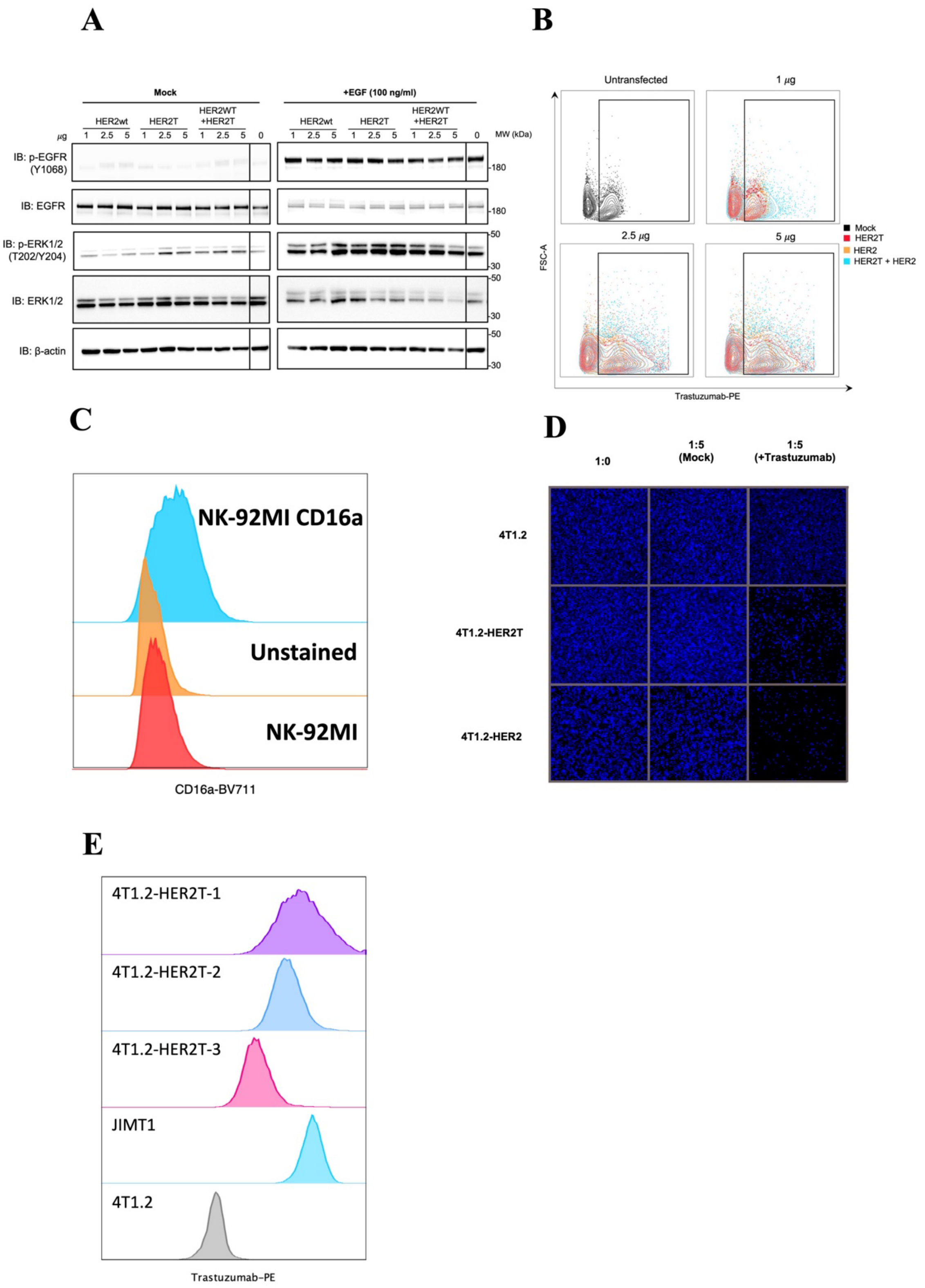
A
Mock
+EGF (100 ng/ml)
HER2wt
HER2T
HER2WT +HER2T
µg
1 2.5 5 1 2.5 5 1 2.5 5 0
MW (kDa)
IB: p-EGFR (Y1068)
IB: EGFR
IB: p-ERK1/2 (T202/Y204)
IB: ERK1/2
IB: β-actin
180
50
30
B
Untransfected
1 µg
2.5 µg
5 µg
FSC-A
Trastuzumab-PE
Mock
HER2T
HER2
HER2T + HER2
C
NK-92MI CD16a
Unstained
NK-92MI
CD16a-BV711
D
1:0
1:5 (Mock)
1:5 (+Trastuzumab)
4T1.2
4T1.2-HER2T
4T1.2-HER2
E
4T1.2-HER2T-1
4T1.2-HER2T-2
4T1.2-HER2T-3
JIMT1
4T1.2
Trastuzumab-PE

Supplementary Figure S1. (A) 786-O cells were transfected with different amounts of HER2 or HER2T cDNA, or both, as indicated. 24 hours post-transfection, cells were serum starved for 24 hours, and subsequently stimulated with or without EGF (100 ng/ml) for 30 minutes, then harvested for protein extraction. Whole cell lysates were resolved by SDS-PAGE and probed with the indicated antibodies. **(B)** 786-O cells from (A) were analyzed by flow cytometry for HER2 or HER2T expression. **(C)** NK-92MI CD16a+ cells were analyzed by flow cytometry for CD16a expression. **(D)** Target cells were incubated with or without trastuzumab, in the presence or absence of NK92-MI CD16a+ cells at 1:5 T:E ratio. 12-16 hours post-coculture, wells were washed to remove NK cells, and remaining target cells were stained with DAPI and imaged using the Cellomics Arrayscan (5x magnification). **(E)** 4T1.2-HER2T clones and control cells were analyzed by flow cytometry for HER2 or HER2T levels, using trastuzumab as a primary antibody.

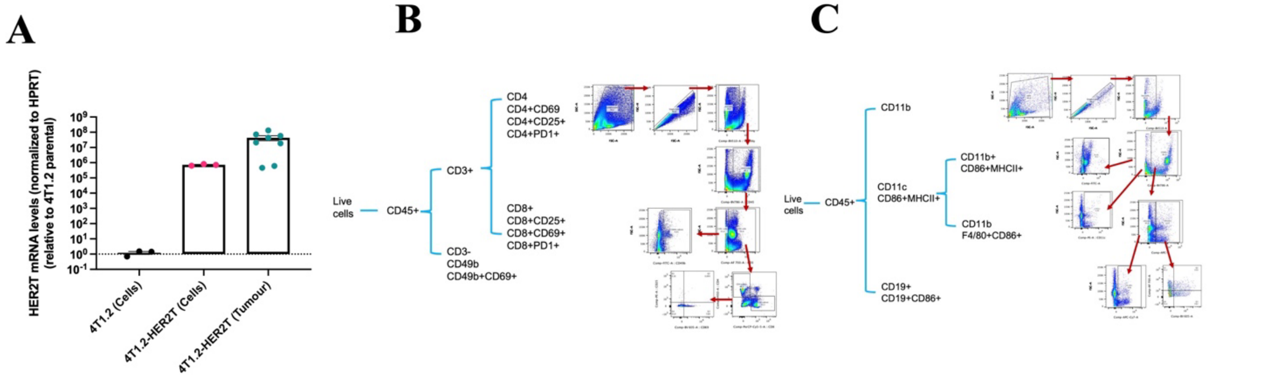

D

Tumour				
	4T1.2 vs 4T1.2-HER2T (s.c.)	4T1.2 vs 4T1.2-HER2T (o.t.)	4T1.2 (s.c. vs o.t.)	4T1.2-HER2T (s.c. vs o.t.)
CD4+	>0.9999	0.9997	0.9998	0.9932
CD4+CD25+	>0.9999	>0.9999	>0.9999	>0.9999
CD8+	>0.9999	>0.9999	>0.9999	0.9997
CD8+CD25+	>0.9999	>0.9999	>0.9999	>0.9999
CD3-CD49b+	0.1378	0.9997	**0.0002**	**0.0067**
CD3-CD49+CD69+	0.9998	0.9463	0.9705	0.9974
CD11b+F4/80+	0.8997	0.9863	0.2126	0.3582
CD11b+F4/80+CD86+	0.814	>0.9999	0.2265	0.3763
CD11c+	0.9248	0.1634	0.9308	0.9671
CD11c+CD86+	0.9958	0.5892	0.9477	0.9964
CD11c+MHCII+CD86+	0.9966	0.5187	0.9418	0.9873
CD19+	0.9960	0.9382	0.9892	0.9617
CD19+CD86+	>0.9999	0.9507	0.9867	>0.9999

E

Lymph nodes				
	4T1.2 vs 4T1.2-HER2T (s.c.)	4T1.2 vs 4T1.2-HER2T (o.t.)	4T1.2 (s.c. vs o.t.)	4T1.2-HER2T (s.c. vs o.t.)
CD4+	**<0.0001**	0.3204	0.6601	**<0.0001**
CD4+CD25+	>0.9999	0.9981	>0.9999	0.9798
CD8+	0.8683	0.9992	0.7176	**<0.0001**
CD8+CD25+	>0.9999	>0.9999	>0.9999	>0.9999
CD3-CD49b+	0.9965	0.9974	0.9872	0.7237
CD3-CD49+CD69+	>0.9999	>0.9999	>0.9999	0.9985
CD11b+F4/80+	0.9987	0.9693	0.9777	0.928
CD11b+F4/80+CD86+	0.9989	0.9771	0.9821	0.9338
CD11c+	0.9957	0.9998	0.9754	0.4253
CD11c+CD86+	0.9996	>0.9999	0.9992	0.933
CD11c+MHCII+CD86+	0.9996	>0.9999	0.999	0.9145
CD19+	0.1641	0.5703	0.6974	**<0.0001**
CD19+CD86+	0.9564	0.9293	0.9772	0.6714

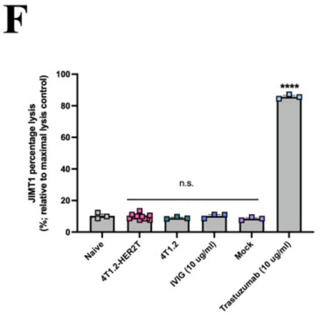

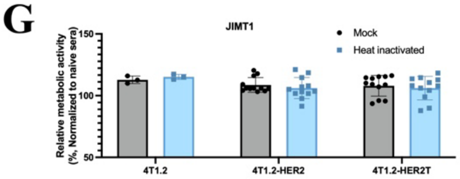

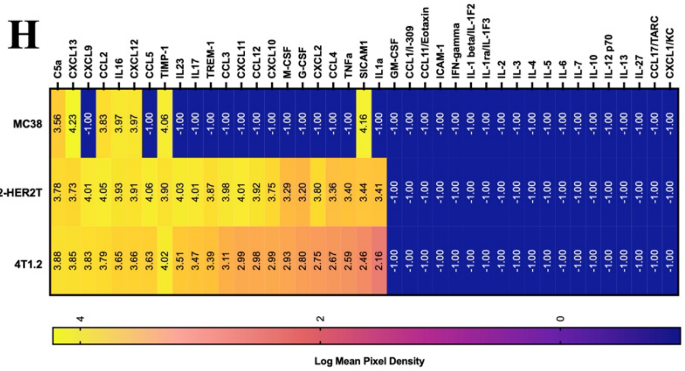

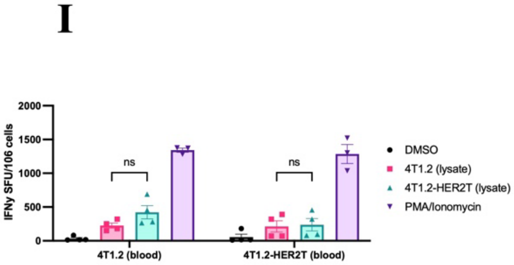

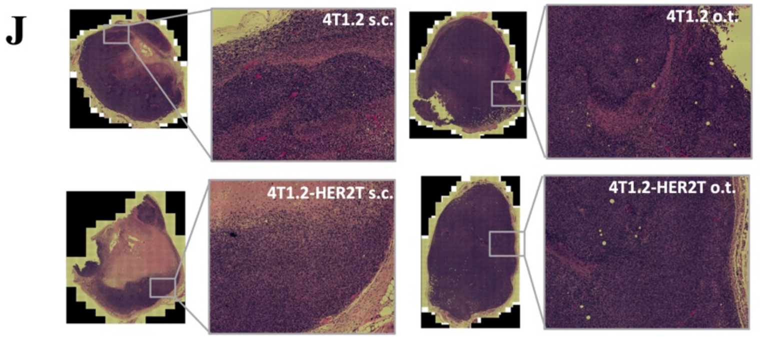

Supplementary Figure S2. (A) RNA was extracted from cells or tumours as indicated. HER2T mRNA was assessed by qRT-PCR, and normalized to *HPRT,* and graphed relative to 4T1.2 expression levels. (mean ± SEM, 4T1.2 n=3, 4T1.2-HER2T cells n=2, 4T1.2-HER2T s.c. tumours n=8, CT26wt-HER2T s.c. tumours n=9). **(B-C)** Flow cytometry gating strategy used during profiling of baseline immune profile of the 4T1.2-HER2T tumour model (s.c. and o.t.) for **(B)** T cells or NK cells, or **(C)** antigen-presenting cells (APCs). **(D-E)** p-values for the comparisons between groups in Figure 3E–H; 2-way ANOVA with Tukey's correction for multiple comparisons. Significant comparisons bolded. **(F)** JIMT1-NLuc cells were incubated with serum collected from naïve mice, or tumour bearing mice (4T1.2-HER2T or 4T1.2), or incubated with IVIS, mock, or trastuzumab at the indicated concentrations. Following washes, JIMT1-NLuc cells were co-cultured with NK92-MI CD16a cells for 12 hours. Levels of nanoluciferase release was measured in the supernatant following co-culture, and normalized to maximum lysis control. (mean ± SEM; Naive n=3, 4T1.2-HER2T n=12, 4T1.2 n=3, IVIG n=3, Mock n=3, Trastuzumab n=3; **** $p < 0.0001$ relative to Naïve control; one-way ANOVA with Dunnet's test for multiple comparison;) **(G)** JIMT1 cells were incubated with fresh or heat-inactivated serum (1:100) from tumour-bearing mice for 24 hours (4T1.2 n=3, 4T1.2-HER2 n=12, 4T1.2-HER2T n=12). Cell viability was assessed by AlamarBlue (mean ± SEM, 4T1.2 n=3, 4T1.2-HER2 n = 12, 4T1.2-HER2T n=12) **(H)** Serum cytokine/chemokine profile from tumour bearing mice. **(I)** IFNγ ELISPOT results from murine PBMCs isolated from tumour-bearing mice (mean ± SEM; 4T1.2 n=2, 4T1.2-HER2T n=4; n.s. one-way ANOVA with Tukey's correction for multiple comparison,) mice and stimulated with 4T1.2 or 4T1.2-HER2T lysates (or PMA/ionomycin control). **(J)** 4T1.2 s.c./o.t. and 4T1.2-HER2T s.c./o.t. tumours were harvested, fixed, sectioned, and stained by H&E. Tissue slides were scanned at 20x magnification using the ZEISS Axio Scan.Z1 and assessed by a pathologist. (Summary of pathology report: In all tumours, atypical, hyperchromatic and pleomorphic cells were observed, with notable background of acute and chronic inflammation and coagulative necrosis. Immune infiltration was noted, especially by eosinophils, with irregular invasions into lympho-vascular structures. In subcutaneous tumours, distinct hypercellular nodules were noted, with sections overall containing a greater extent of coagulative geographic necrosis compared with mammary fat pad tumours. All features observed were in keeping with malignancy, with no distinguishing characteristics between HER2T+ and wildtype tumours.)

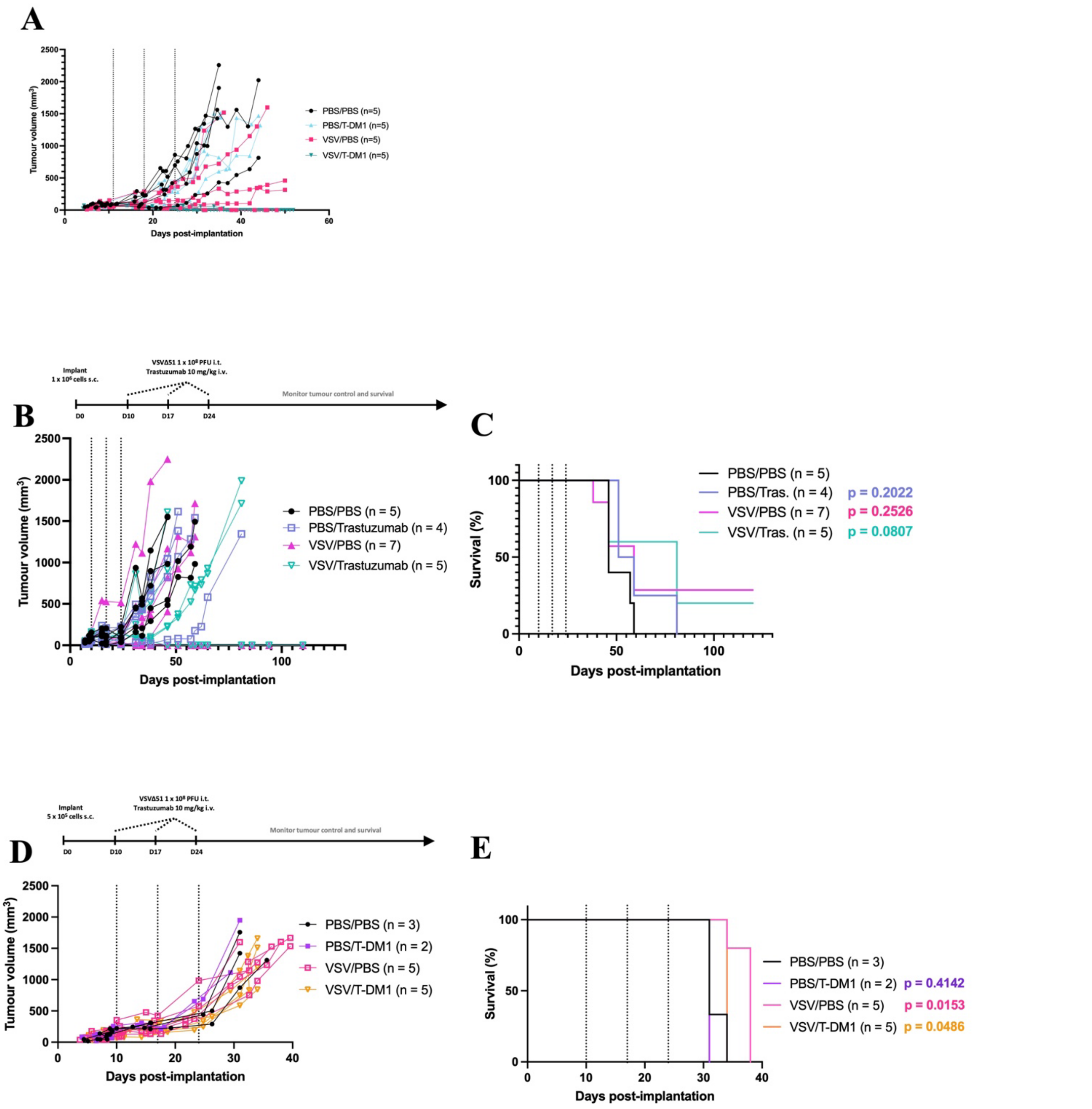
A
Tumour volume (mm³)
Days post-implantation
PBS/PBS (n=5)
PBS/T-DM1 (n=5)
VSV/PBS (n=5)
VSV/T-DM1 (n=5)
B
Implant
1 x 10⁶ cells s.c.
VSVΔ51 1 x 10⁸ PFU i.t.
Trastuzumab 10 mg/kg i.v.
Monitor tumour control and survival
D0
D10
D17
D24
Tumour volume (mm³)
Days post-implantation
PBS/PBS (n = 5)
PBS/Trastuzumab (n = 4)
VSV/PBS (n = 7)
VSV/Trastuzumab (n = 5)
C
Survival (%)
Days post-implantation
PBS/PBS (n = 5)
PBS/Tras. (n = 4) p = 0.2022
VSV/PBS (n = 7) p = 0.2526
VSV/Tras. (n = 5) p = 0.0807
D
Implant
5 x 10⁵ cells s.c.
VSVΔ51 1 x 10⁸ PFU i.t.
Trastuzumab 10 mg/kg i.v.
Monitor tumour control and survival
D0
D10
D17
D24
Tumour volume (mm³)
Days post-implantation
PBS/PBS (n = 3)
PBS/T-DM1 (n = 2)
VSV/PBS (n = 5)
VSV/T-DM1 (n = 5)
E
Survival (%)
Days post-implantation
PBS/PBS (n = 3)
PBS/T-DM1 (n = 2) p = 0.4142
VSV/PBS (n = 5) p = 0.0153
VSV/T-DM1 (n = 5) p = 0.0486

Supplementary Figure S3. (A) Individual mouse tumour volumes following treatment as in Figure 6B. **(B-C**) BALB/c mice were implanted with 4T1.2-HER2T s.c. tumours and treated as indicated (n=5 per group). (B) Tumour volumes and (C) survival were monitored. **(D-E)** BALB/c mice were implanted with 4T1.2 parental s.c. tumours and treated as indicated (n=5 per group). (D) Tumour volumes and (E) survival were monitored. The dotted lines represent treatments.

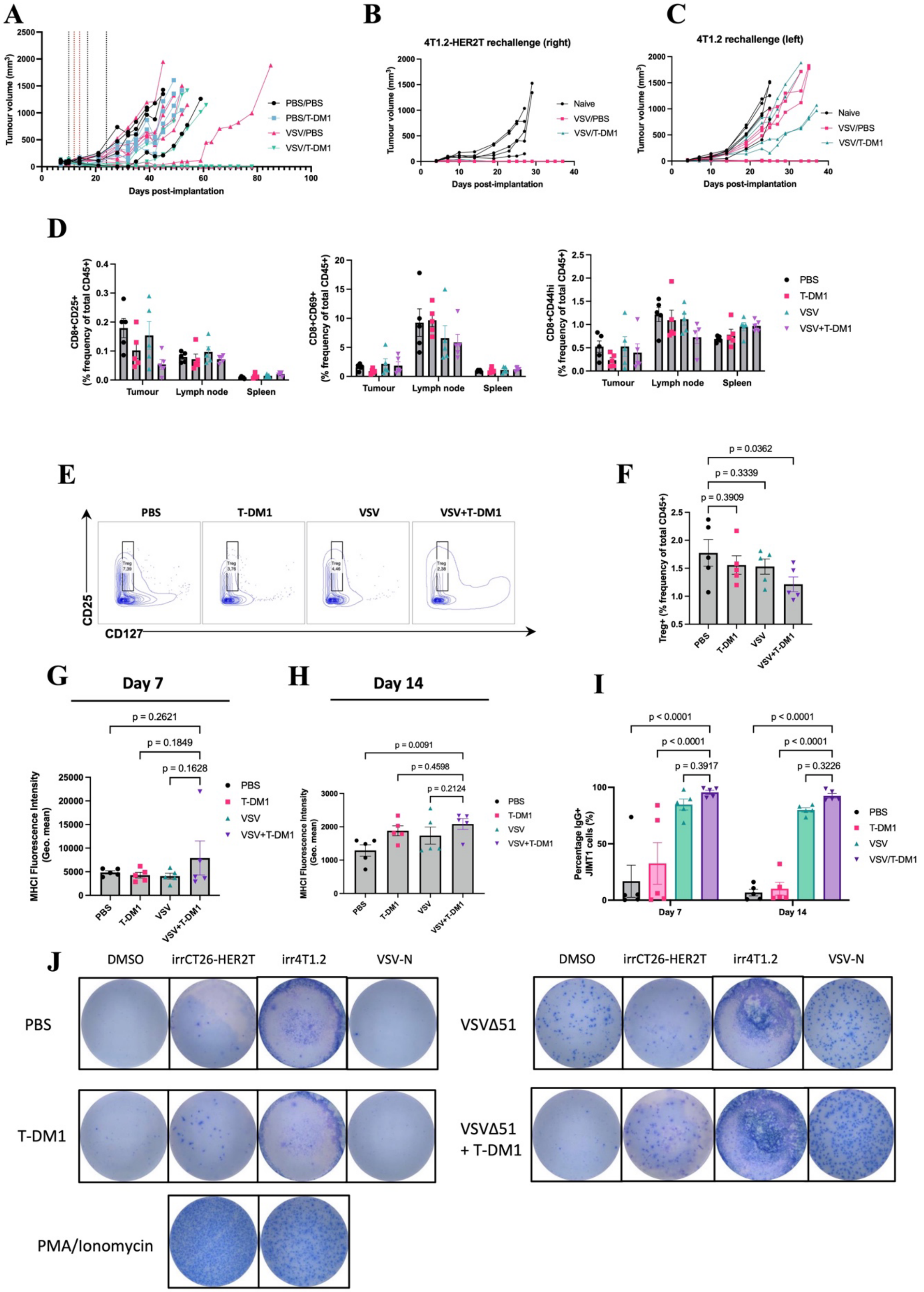
A
B
4T1.2-HER2T rechallenge (right)
C
4T1.2 rechallenge (left)
Tumour volume (mm³)
Days post-implantation
PBS/PBS
PBS/T-DM1
VSV/PBS
VSV/T-DM1
Naive
D
CD8+CD25+ (% frequency of total CD45+)
CD8+CD69+ (% frequency of total CD45+)
CD8+CD44hi (% frequency of total CD45+)
Tumour
Lymph node
Spleen
PBS
T-DM1
VSV
VSV+T-DM1
E
CD25
CD127
F
Treg+ (% frequency of total CD45+)
p = 0.0362
p = 0.3339
p = 0.3909
G
Day 7
MHCI Fluorescence Intensity (Geo. mean)
p = 0.2621
p = 0.1849
p = 0.1628
H
Day 14
p = 0.0091
p = 0.4598
p = 0.2124
I
Percentage IgG+ JIMT1 cells (%)
p < 0.0001
p = 0.3917
p = 0.3226
Day 7
Day 14
J
DMSO
irrCT26-HER2T
irr4T1.2
VSV-N
PBS
T-DM1
PMA/Ionomycin
VSVΔ51
VSVΔ51 + T-DM1

Supplementary Figure S4. (A) Individual mouse tumour volumes from Figure 5B. **(B-C)** Individual mouse tumour volumes from the rechallenge experiment in Figure 5D–E. **(D)** Levels of CD8+ T-cell activation within tumours, draining lymph nodes, and spleens, at day 7 post-treatment. **(E-F)** Levels of T-reg in tumour draining lymph nodes at day 7. **(G-H)** Levels of MHC class I on tumour cells at (G) day 7 and (H) day 14 post-treatment. (mean ± SEM, n=5 per group; p-value calculated by one-way ANOVA with Fisher's LSD test). **(I)** Percentage of serum-bound JIMT1 cells from Figure 7N. (mean ± SEM, n=5 per group; p-value calculated by two-way ANOVA with Fisher's LSD test). **(J)** Representative ELISpot images for Figure 7O-Q.

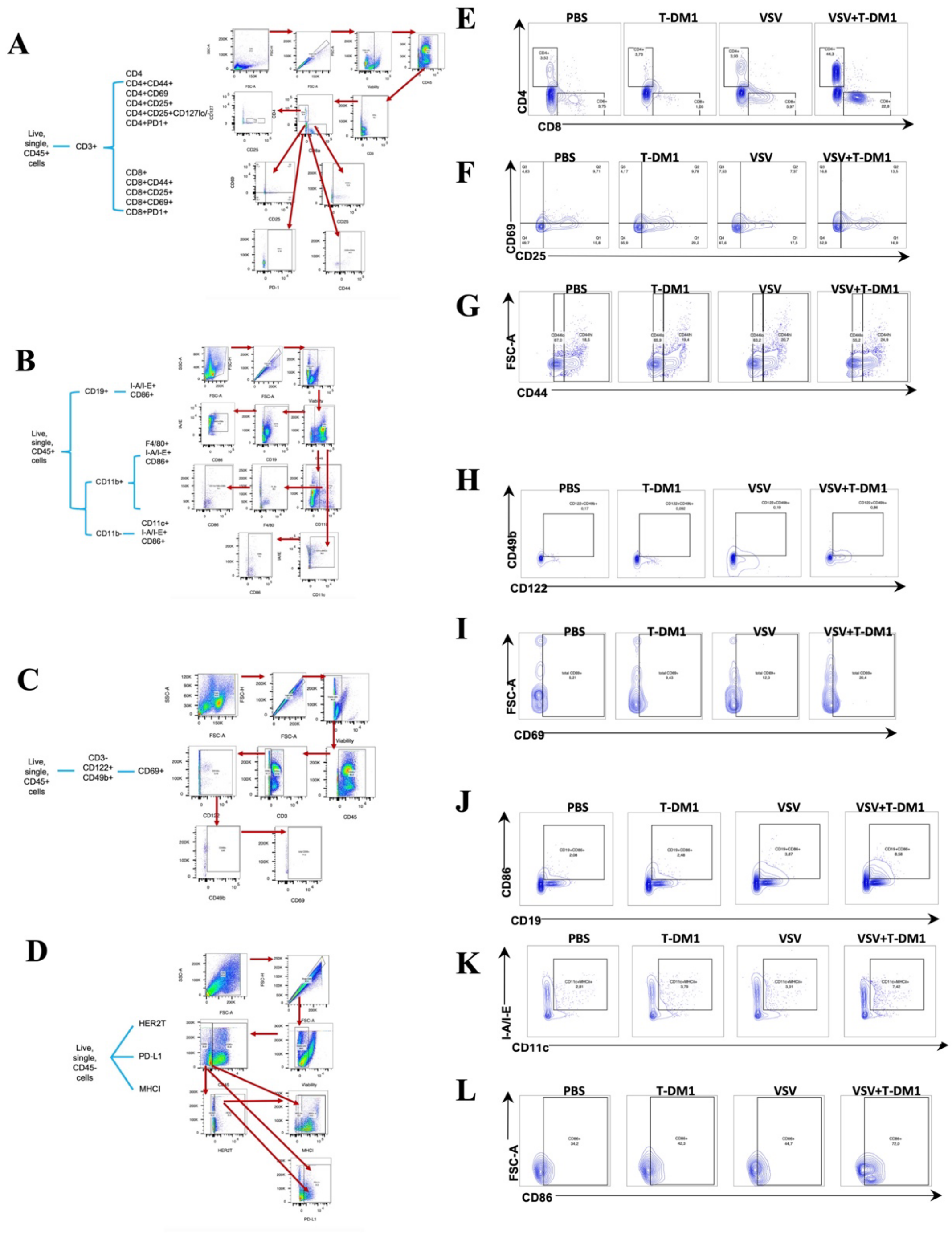
A
Live, single, CD45+ cells
CD3+
CD4
CD4+CD44+
CD4+CD69
CD4+CD25+
CD4+CD25+CD127lo/-
CD4+PD1+
CD8+
CD8+CD44+
CD8+CD25+
CD8+CD69+
CD8+PD1+
B
Live, single, CD45+ cells
CD19+
I-A/I-E+
CD86+
CD11b+
F4/80+
I-A/I-E+
CD86+
CD11b-
CD11c+
I-A/I-E+
CD86+
C
Live, single, CD45+ cells
CD3-
CD122+
CD49b+
CD69+
D
Live, single, CD45- cells
HER2T
PD-L1
MHCI
E
PBS
T-DM1
VSV
VSV+T-DM1
CD4
CD8
F
CD69
CD25
G
FSC-A
CD44
H
CD49b
CD122
I
FSC-A
CD69
J
CD86
CD19
K
I-A/I-E
CD11c
L
FSC-A
CD86

Supplementary Figure S5. Flow gating strategies for **(A)** T-cells, **(B)** antigen-presenting cells, **(C)** NK cells, **(D)** tumours. Representative contour plots from **(E)** Figure 7H total CD4+ and CD8+ T-cells, **(F-G)** Figure 7I activated CD4+ T-cells, **(H-I)** Figure 7J total or activated NK cells, (**J)** Figure 7K, total and activated B-cells, **(K-L)** Figure 7M, total and activated dendritic cells.
